# Quantitative trait loci mapping of polyphenol metabolites from a ‘Latham’ x ‘Glen Moy’ red raspberry (*Rubus idaeus* L) cross

**DOI:** 10.1007/s11306-023-02033-7

**Published:** 2023-08-08

**Authors:** G. J. McDougall, J. W. Allwood, G. Dobson, C. Austin, S. Verrall, C. J. Alexander, R. D. Hancock, J. Graham, C. A. Hackett

**Affiliations:** 1 Plant Biochemistry and Food Quality Group, Environmental and Biochemical Sciences Department, The James Hutton Institute, Dundee, DD2 5DA Scotland, UK; 2 Ecological Sciences Department, The James Hutton Institute, Dundee, DD2 5DA Scotland, UK; 3 Biomathematics and Statistics Scotland (BioSS), The James Hutton Institute, Dundee, DD2 5DA Scotland, UK; 4 Cell and Molecular Sciences Department, The James Hutton Institute, Dundee, DD2 5DA Scotland, UK

**Keywords:** Raspberry, Polyphenols, Anthocyanins, Ellagitannins, Genetic control, Heritability, QTLs

## Abstract

**Objective:**

The objective of this study was to investigate the genetic control of polyphenol accumulation in red raspberry (*Rubus idaeus* L).

**Methods:**

The levels of total anthocyanins and 37 individual polyphenol metabolites were measured over three years in a raspberry biparental mapping population. Quantitative trait loci (QTLs) for these traits were mapped onto a high-density SNP linkage map.

**Results:**

At least one QTL was detected for each trait, with good consistency among the years. On four linkage groups (LG), there were major QTLs affecting several metabolites. On LG1, a QTL had large effects on anthocyanins and flavonols containing a rutinoside or rhamnose group. On LG4, a QTL had large effects on several flavonols and on LG5 and LG6 QTLs had large effects on ellagic acid derivatives. Smaller QTLs were found on LG2 and LG3.

**Conclusion:**

The identification of robust QTLs for key polyphenols in raspberry provides great potential for marker-assisted breeding for improved levels of potentially health beneficial components.

**Supplementary Information:**

The online version contains supplementary material available at 10.1007/s11306-023-02033-7.

## Introduction

World raspberry production has risen steadily from 520 000 tonnes in 2010 to 896 000 tonnes in 2020 [FAO figures] with 75% of production in Europe. The value of raspberry production quadrupled in the UK from 1995 to 2015 supported by a drive for increased class I cultivation for the fresh market [DEFRA figures] although class II fruits also supply the increasing popularity of raspberry juice products (Rajauria & Tiwari, 2018) as well as traditional uses in purees, jams, and preserves.

Raspberries are valued for their distinctive flavour and colour and raspberry fruit breeding has widened its focus from traits associated with agronomic performance and disease resistance towards those associated with fruit sensory quality (Jennings, 2018; Jennings et al., 2016) and potential health benefits (e.g., Mazzoni et al., 2016). The potential health benefits of raspberries have been associated with their polyphenol content and composition (e.g., Hancock et al., 2018). The distinctive coloration of red raspberries is due to their accumulation of anthocyanins during ripening, but these are accompanied by the presence of ellagitannins, ellagic acid derivatives, and flavonols (e.g., Carvalho et al., 2013; McDougall et al., 2005, 2014; Mullen et al., 2003). The anthocyanins and ellagitannins are the major polyphenol components and have received the most attention for potential health benefits (e.g. Rao & Snyder, 2010). The ellagitannins, in particular, contribute to astringency, which is an important factor in the characteristic sensory properties of this fruit (e.g. He et al., 2015).

The levels of anthocyanins, ellagitannins and other phenolics vary between different Rubus species and indeed different raspberry varieties (e.g. Bradish et al., 2012; Deighton et al., 2000). Although phenolic content and composition can vary with agronomic practice, location of growth, abiotic and biotic stresses, there is a strong genetic element in the control of accumulation (Anttonen & Karjalainen, 2005; Connor et al. 2005; Remberg et al., 2010; Dobson et al., 2012; Bradish et al., 2012).

Previous studies have used the genetic linkage map developed from a cross between red raspberry cultivars, Glen Moy and Latham (Graham et al., 2004), followed by Quantitative Trait Loci (QTL) mapping and have identified multiple markers for a broad range of important raspberry agronomical characteristics, which can lead to improved cultivars [e.g. McCallum et al., 2018; Scolari et al., 2021]. Also using this approach, studies at the James Hutton Institute established QTLs for total anthocyanin content and for the levels of selected major individual raspberry anthocyanins (Kassim et al., 2009; McCallum et al., 2010; Paterson et al., 2013). In other studies, consistent QTLs were also established for total phenol content and total anthocyanin content in year-on-year studies (Dobson et al, 2012). As total anthocyanin content is a sub-set of total phenol content, QTLs for total phenol which did not align with those for total anthocyanin content could be related to variation in the other polyphenol components.

The genetic linkage map used in these studies based on the Glen Moy-Latham cross has subsequently been enhanced using GbS (Hackett et al., 2018), providing a higher-density GbS map that allows QTLs to be identified more precisely. The establishment of a draft genome sequence for Glen Moy aided the development of the GbS map and also allows direct assignment of SNP marker information to the Glen Moy genome scaffolds. These resources provide the ability to perform more precise QTL analysis and genes associated with QTLs can also be examined against gene expression data across fruit development to correlate expression against the timing of polyphenol accumulation. This study examines the variation in individual polyphenol components in the progeny set of this cross identified by liquid chromatography mass spectrometry (LC–MS^n^) across three years of cultivation. We combine this data with marker data to identify robust and consistent QTLs associated with specific polyphenol classes. The data presented offers opportunities for the development of markers to accelerate the development of raspberry varieties with specific increases in key polyphenolic antioxidants.

## Materials and methods

### Mapping population and map

The mapping population was the previously reported cross between the North American raspberry Latham and the Scottish raspberry Glen Moy, with 188 offspring (Graham et al. 2004). This cross has been studied in detail over many years at the James Hutton Institute, for traits such as fruit ripening and fruit characteristics including anthocyanin content (e.g. Graham et al., 2009; Kassim et al., 2009; McCallum et al., 2010). These traits have been mapped on a map of medium density, with up to 439 markers for the study of crumbly fruit by Graham et al. (2015). More recently the linkage map was extended by 2348 SNPs from genotyping-by-sequencing to give a high-density linkage map and the fruit ripening data was reanalysed using this map (Hackett et al., 2018). The high-density map has been used for the QTL interval mapping analysis below.

### Metabolite analysis using liquid chromatography mass spectrometry (LC-MS^n^)

Fruit from the parents and offspring of the mapping population were analysed for metabolite profiles over 3 years, 2010, 2011 and 2015. In 2010, two field replicates of the population were analysed and in 2011 and 2015, three field replicates were used for each year. The analysis was carried out using LC–MS^n^, with one batch for each field replicate in 2010 and 2011. In 2015, extra quality control samples were included, and the samples were analysed in five batches. The weather conditions in the analysis years, 2010, 2011 and 2015 were substantially different in maximum temperature, distribution of rainfall and sun hours across the growing season (results not shown).

As before (Dobson et al., 2012), fruit was picked when ripe, placed in labelled bags stored on ice, transferred to the laboratory within one hour and frozen at − 20 °C until extraction. Whilst still frozen, a representative subsample of fruit from each progeny (5–8 berries equivalent to 7–10 g) was weighed. The selected berries were cut in half, then extracted using a glass tissue homogenizer with a PTFE pestle with an equal volume to weight of ice-cold 100% acetonitrile (ACN) containing 0.1% formic acid (FA). This method does not crush the seeds, so it is possibly not representative of some components (e.g., ellagitannins) that are known to be enriched in the seeds (e.g., Arnold et al., 2022). The homogenate was transferred in a centrifuge tube and centrifuged at 2 750 g for 10 min at 5 °C (Eppendorf 5810 R) and the supernatant aliquoted into 1 mL amounts. The extracts were analysed for total anthocyanin content as described by McDougall et al. (2005) using a standard curve of cyanidin-3-*O*-glucose (Extrasynthese, Genay, France). The aliquots were dried in a Speed-Vac then stored at − 20 °C.

The dried raspberry extracts were resuspended in 475 μL of 10% ACN containing 0.1% FA with vortex mixing then 25 μL of internal standard solution (0.5 mg/mL morin in methanol; Sigma Chem Co. Ltd) was added. After centrifugation at 10 000 × g for 10 min at 5 °C, the supernatants were removed and placed in 0.45 μM PTFE filter vials (Thomson Instrument Company, Bioprocess Engineering Services Ltd, Kent, UK) prior to analysis. Samples were analysed using a LC system consisting of an Accela 600 quaternary pump and Acela PDA detector coupled to an LTQ Orbitrap mass spectrometer (Thermo Fisher Scientific Ltd.). Samples (10 μL) were injected onto a 2 × 150 mm (4 μm) Synergy Hydro-RP 80 fitted with a C18 4 × 2 mm Security Guard cartridge (Phenomenex Ltd, Macclesfield, UK.). Auto-sampler and column temperatures were maintained at 6 and 30 °C, respectively. The samples were analysed at a flow rate of 200 μL/min using a binary mobile phase of (A) 0.1% aqueous formic acid and (B) 0.1% formic acid in 50% acetonitrile/water with the following gradient: 0−5 min, 5% B; 5−22 min, 5−50% B; 22−32 min, 50−100% and 32–34 min 100% B. Mass detection was carried out using an LTQ Orbitrap mass spectrometer in positive ESI mode. Two scan events were employed; full-scan analysis was followed by data-dependent MS/MS of the three most intense ions using collision energies of 45 eV source voltage (set at 3.4 kV) in wide-band activation mode. The instrument was optimized by tuning against morin at a resolution of 100 000 in a range of 80−2000 mass units. For optimal electrospray ionization, the source conditions were set at a source temperature of 280 °C, sheath gas at 60 arbitrary units, and an auxiliary gas at 5 arbitrary units. Prior to analysis, the mass accuracy of the instrument was assured by calibration following the manufacturer’s protocols. All predicted formula data presented were accurate at < 2 ppm.

The samples were analysed in a randomised order and the quality of the MS response was checked by monitoring blanks containing the internal standard and quality control samples of samples from the parental lines interspersed through the sequence of samples. After peak checking, raw peak areas for the major components were obtained using the resident Xcalibur software. After export of MS peak data to Microsoft Excel, the peak areas were ratioed against the internal standard (morin).

Metabolites were identified by their MS properties, the formulae derived from the exact mass data and fragmentation MS^2^ data and compared against literature on raspberry components as discussed. Only cyanidin-3-*O*-glucoside, pelargonidin-3-*O*-glucoside, cyanidin-3-*O*-sophoroside, quercetin-3-*O*-glucoside and ellagic acid were confirmed against standard compounds (Extrasynthese Ltd).

### Statistical analysis

#### Exploratory analysis

Each metabolite was analysed using a linear mixed model, fitted using REML (GenStat 20, VSN International, 2019). The metabolites were transformed using a log_10_ transformation before analysis to stabilise the variance. To estimate genotype means for QTL mapping, data from each year was first analysed separately and genotype was treated as a fixed effect. For 2015, a random effect of batch in addition to that of field replicate was tested but was not significant. A mean over the years was also estimated from a mixed model with genotype, year and their interaction as fixed effects and field replicate within year as a random effect. The generalised heritabilities of the genotypes and the genotype × year interactions were estimated using the GenStat VHERITABILITY procedure. For this analysis, genotype, year, and field replicate within year were treated as random effects. Pearson correlations between the metabolites were calculated for each year separately, based on the log-transformed genotype means.

#### QTL analysis

For each year separately, and for the mean across years, QTL interval mapping was carried out for each metabolite using the high-density SNP linkage map and the mapping approach from Hackett et al. (2018), based on a hidden Markov model (HMM) to estimate genotype probabilities. This method was shown in that paper to give better peak resolution than software such as MapQTL and GenStat for this population, where there is much more information about markers from the Latham parent than from Glen Moy. The analysis combines information across genetic markers along the chromosome to estimate the probabilities of each possible QTL genotype for each offspring at each position. In a cross such as this with outbreeding parents, the parental genotypes at a QTL are usually represented as ab × cd, with offspring genotypes ac, ad, bc, and bd. Genetic predictors for the Latham additive effect (*P*_*1*_*)*, the Glen Moy additive effect (*P*_*2*_) and the dominance effect (*D*) can then be derived from the genotype probabilities *pr(ac)* etc. as:

$${P}_{1}=pr(bc)+pr(bd)-pr(ac)-pr(ad)$$

$${P}_{2}=pr(bd)+pr(ad)-pr(bc)-pr(ac)$$

$$D=pr(bd)-pr(bc)-pr(ad)+pr(ac)$$

A permutation study was carried out on this population for highly multivariate imaging data (Williams et al., 2021) and estimated the genome-wide 95% LOD threshold as 3.86; the same threshold has been used for all traits here. A thinned SNP map was used to carry out a QTL × year analysis in GenStat, with a stepwise selection of multiple QTLs, as the high-density map was too computationally intensive for this analysis. This used the significance threshold of Li and Ji (2005), with a genome-wide significance of 0.05.

## Results and discussion

### LC–MS^n^ analysis of polyphenol metabolites

Thirty-seven metabolites were detected in all 3 years (Table 1). These comprised 12 anthocyanins, ten of which could be putatively identified from previous work on red raspberries (Bradish et al., 2012; McDougall et al., 2005; Mullen et al., 2003) i.e. cyanidin sophoroside, cyanidin glucoside, cyanidin sophoroside rhamnose /cyanidin glucosyl rutinoside, cyanidin sambubioside, cyanidin rutinoside/pelargonidin sophoroside, cyanidin sambubioside rhamnoside/cyanidin xylosyl rutinoside, pelargonidin glucoside, pelargonidin glucosyl rutinoside, pelargonidin sambubioside and pelargonidin rutinoside). Three of these anthocyanin components could not be distinguished by their molecular formulae or their MS^2^ properties so are labelled with both possible structures (e.g. CyRut/PelSoph). Two other anthocyanin-like components (CyC_28_H_30_O_17_ and CyC_34_H_40_O_21_) were identified as possible cyanidin derivatives due to their fragmentation to *m/z* 287 [which is consistent with cyanidin (Cy)] and were named with their putative molecular formula. Four major ellagitannins were noted (Lambertianin C, Sanguiin H2, Sanguiin H6 and Sanguiin H10) and five ellagic acid derivatives (ellagic acid, ellagic acid acetyl pentose, ellagic acid pentose 1, ellagic acid pentose 2, and methyl ellagic acid pentose) (Gasperotti et al., 2010; McDougall et al., 2014). As the homogenisation method did not crush the seeds, the metabolic profile probably more reflects the flesh of the raspberries and may not capture the entirety of ellagitannin constituents, which are known to be enriched in the seeds (Arnold et al., 2022). However, the extractable components should be consistent across the progeny. Sixteen flavonol derivatives were noted (i.e. kaempferol glucuronide, galactoside and glucoside but also quercetin dihexosides, glucuronide, galactoside, glucoside, hexosyl rhamnosides, glucosyl rutinosides, pentose and malonyl glucoside). The putative identification of these components was also aided by SOPs delivered through the EUBERRY project (Dobson et al., 2010).

**Table 1:** Components identified in raspberry progeny

Peak	RT	PDA max	*m/z* [M + H]^+^	MS^2^	Predicted formula [M + H]^+^	Putative identity	Short name
A1	4.23	515	611.1603	287	C_27_H_31_O_16_	Cyanidin3-*O*-sophoroside^a^	CySoph
A2	4.52	515	449.1075	287	C_21_H_21_O_11_	Cyanidin-3-*O*-glucoside^a^	CyGlu
A3	4.62	515	757.2184	287, **611**	C_33_H_41_O_20_	Cyanidin sophoroside rhamnoside/ Cyanidin glucosyl rutinoside	CySophRham/ CyGluRut
A4	4.69	515	581.1503	287	C_26_H_29_O_15_	Cyanidin sambubioside	CySam
A5	4.94	505	639.1566	287	C_28_H_31_O_17_	Cyanidin derivative	CyC_28_H_30_O_17_
A6	4.97	510	595.1660	271, **287**, 449	C_27_H_31_O_15_	Cyanidin rutinoside/ Pelargonidin sophoroside	CyRut/PelSoph
A7	5.05	510	727.2079	**287**, 433, 581	C_32_H_39_O_19_	Cyanidin sambubioside rhamnoside/ Cyanidin xyloside rutinoside	CyXylRut/ CySamRham
A8	5.22	510	433.1127	271	C_21_H_21_O_10_	Pelargonidin-3-*O*-glucoside^a^	PelGlu
A9	5.26	510	785.2134	**287**, 639	C_34_H_41_O_21_	Cyanidin derivative	CyC_34_H_40_O_21_
A10	5.44	510	741.2240	**271**, 417, 595	C_33_H_41_O_19_	Pelargonidin sophoroside rhamnoside/ Pelargonidin glucosyl rutinoside	PelGluRut/ PelSophRham
A11	5.76	500	565.1540	ND	C_26_H_29_O_14_	Pelargonidin sambubioside	PelSam
A12	5.73	510	579.1711	**271**, 433	C_27_H_31_O_14_	Pelargonidin rutinoside	PelRut

Peak	RT	PDA max	*m/z* [M − H]^−^	MS^2^	Predicted formula [M − H]^−^	Putative identity	Short name
13	5.98	270	783.0574*	**1265,** 935, 933, 897, 633,** 301**	C_68_H_47_O_44_	Sanguiin H10	Sanguiin H10
14	6.12	355	771.1839	**301**, 591	C_33_H_39_O_21_	Quercetin glucosyl rutinoside	QGluRut1
15	6.34	350	771.1887	301, **591**	C_33_H_39_O_21_	Quercetin glucosyl rutinoside	QGluRut2
16	6.44	350	625.1330	**301**, 445, 505	C_27_H_29_O_17_	Quercetin dihexoside	QDihex1
17	6.60	350	625.1331	301	C_27_H_29_O_17_	Quercetin dihexoside	QDihex2
18	7.14	355	433.0353	301	C_19_H_13_O_12_	Ellagic acid pentoside	EllagicPent1
19	7.43	355	433.0347	301	C_19_H_13_O_12_	Ellagic acid pentoside	EllagicPent2
20	7.22	260	1401.5901*	**633**, 933,1099, 1250, 1567, 1867	C_123_H_79_O_78_	Lambertianin C	Lambertianin
21	7.31	365	300.9947	185, 229, **257**, 284	C_14_H_5_O_8_	Ellagic acid^a^	Ellagic
22	7.47	260	551.0307*	529	C_48_H_31_O_31_	Sanguiin H2	Sanguiin H2
23	7.55	260	1869.3168 (934.059*)	301, 633, 1235, **1567**	C_82_H_53_O_52_	Sanguiin H6	Sanguiin H6
24	7.77	350	595.1219	**301**, 505	C_26_H_27_O_16_	Quercetin hexosylpentoside	QHexPent
25	7.81	355	609.1374	**301**, 463	C_27_H_29_O_16_	Quercetin hexosylrhamnoside	QHexRham1
26	7.98	355	463.0823	301	C_21_H_19_O_12_	Quercetin galactoside	QGal
27	8.15	355	477.0608	301	C_21_H_17_O_13_	Quercetin glucuronide	QGlcU
28	8.17	350	609.1381	**301**, 463	C_27_H_29_O_16_	Quercetin hexosyl rhamnoside	QHexRham2
29	8.28	355	463.0824	301	C_21_H_19_O_12_	Quercetin-3-*O*-glucoside^a^	QGlu
30	8.81	355	433.0713	301	C_20_H_17_O_11_	Quercetin pentoside	QPent
31	8.84	355	447.0866	**285**	C_21_H_19_O_11_	Kaempferol galactoside	KGal
32	8.94	350	549.0812	**505,** 301	C_24_H_21_O_15_	Quercetin malonyl glucoside	QMalGlu
33	8.96	350	447.0511	301, **315**	C_20_H_16_O_12_	Methylellagic acid pentoside	MeEllPent
34	9.09	365	475.0455	301	C_21_H_15_O_13_	Ellagic acid acetyl pentoside	EllAcetylPent
35	9.17	345	461.0656	285	C_21_H_17_O_12_	Kaempferol glucuronide	KGlcU
36	9.18	345	447.0862	285	C_21_H_19_O_11_	Kaempferol glucoside	KGlu
37	9.41	360	463.0812	301	C_21_H_19_O_12_	Quercetin hexoside	QHex

*Doubly charged ion, *m/z* [M-H]^2−^

^a^component confirmed against standard

MS^2^ fragments in bold are the dominant products

Photodiode array (PDA) profiles (280 nm; Fig. S1A) showed the major components in the parents. However, profiles at 520 nm (Fig. S1B) highlighted the anthocyanins with Latham generally showing higher levels of some of the later eluting anthocyanins (A5–A12).

### Exploratory analysis of polyphenol metabolite data

Summary statistics for the metabolite data and for the total anthocyanins are presented in Table 2. No samples from the Glen Moy parent were analysed in 2011. The mixed model analysis showed few statistically significant (p < 0.05) differences between the levels in the parents. For example, CySam and QPent were significantly higher in Glen Moy, whilst CyRut/PelSoph, PelGlu, PelRut, PelGluRut, KGlcU, QGlcU, QGluRut1, QGluRut2 and QDihex1 had significantly higher levels in Latham.

**Table 2:** Summary statistics for metabolite levels and total anthocyanin data in the parents and offspring across the 3 years of harvest

Short name	Year	Latham	Moy	Mean	Min	Max	SD	H_G	H_GY	BM
TA	10	10.481	6.922	8.765	1.924	25.761	3.820	0.593	0.096	N
	11	16.084	NA	9.703	0.000	45.447	5.284			N
	15	8.812	10.093	8.320	1.584	26.799	3.777			N
CySoph	10	7.631	7.195	8.366	1.826	26.167	4.424	0.715	0.181	N
	11	7.301	NA	6.047	0.815	20.078	2.989			N
	15	5.257	6.135	6.151	1.076	15.954	2.508			N
CyGlu	10	2.132	1.950	2.139	0.503	9.710	1.190	0.771	0.089	N
	11	1.421	NA	1.406	0.258	5.012	0.826			N
	15	1.393	1.894	1.643	0.257	4.603	0.856			N
CySophRham/ CyGluRut	10	6.590	2.520	3.364	0.000	16.180	2.999	0.887	0.186	Y
	11	5.014	NA	1.925	0.000	11.318	1.870			Y
	15	2.637	2.579	1.526	0.000	7.150	1.626			Y
**CySam**	10	0.044	**0.252**	0.160	0.022	0.759	0.116	0.799	0.119	N
	11	0.036	NA	0.108	0.000	0.561	0.090			N
	15	0.025	**0.159**	0.109	0.007	0.378	0.079			N
CyC_28_H_30_O_17_	10	0.146	0.197	0.322	0.000	1.465	0.239	0.634	0.066	N
	11	0.086	NA	0.130	0.007	0.574	0.097			N
	15	0.190	0.287	0.320	0.047	1.486	0.196			N
**CyRut/ PelSoph**	10	**3.075**	1.117	1.868	0.114	6.489	1.174	0.830	0.053	N
	11	2.796	NA	1.165	0.056	4.504	0.806			N
	15	**1.840**	1.305	1.025	0.069	5.221	0.748			N
CyXylRut/ CySamRham	10	0.063	0.112	0.095	0.000	0.576	0.101	0.879	0.155	Y
	11	0.042	NA	0.047	0.000	0.417	0.060			Y
	15	0.023	0.078	0.042	0.000	0.301	0.054			Y
**PelGlu**	10	**0.308**	0.063	0.189	0.014	1.406	0.195	0.795	0.205	N
	11	0.264	NA	0.123	0.000	1.512	0.179			N
	15	**0.224**	0.148	0.144	0.010	0.975	0.130			N
CyC_34_H_40_O_21_	10	0.129	0.065	0.131	0.000	0.646	0.136	0.885	0.109	Y
	11	0.051	NA	0.038	0.000	0.343	0.049			Y
	15	0.099	0.107	0.081	0.000	0.978	0.106			Y
**PelGluRut**	10	**1.807**	0.149	0.350	0.000	3.078	0.397	0.891	0.135	Y
	11	1.965	NA	0.239	0.000	1.979	0.310			Y
	15	**1.066**	0.218	0.166	0.000	1.582	0.224			Y
PelSam	10	0.004	0.002	0.010	0.000	0.139	0.016	0.718	0.227	Y
	11	0.000	NA	0.004	0.000	0.113	0.012			Y
	15	0.005	0.005	0.007	0.000	0.053	0.008			N
**PelRut**	10	**0.221**	0.030	0.078	0.000	0.514	0.091	0.880	0.099	Y
	11	0.155	NA	0.035	0.000	0.587	0.060			Y
	15	**0.123**	0.082	0.038	0.000	0.554	0.063			Y
SanguiinH10	10	0.294	0.722	0.397	0.154	1.000	0.114	0.572	0.046	N
	11	0.104	NA	0.131	0.037	0.352	0.055			N
	15	0.225	0.309	0.301	0.073	0.854	0.112			N
**QGluRut1**	10	**0.074**	0.023	0.016	0.000	0.149	0.021	0.847	0.134	Y
	11	0.065	NA	0.012	0.000	0.073	0.016			Y
	15	**0.081**	0.045	0.023	0.000	0.171	0.031			Y
**QGluRut2**	10	**0.158**	0.070	0.064	0.000	0.491	0.070	0.894	0.164	Y
	11	0.136	NA	0.033	0.000	0.201	0.036			Y
	15	**0.119**	0.073	0.048	0.000	0.301	0.057			Y
QDihex1	10	**0.097**	0.015	0.022	0.000	0.156	0.026	0.861	0.139	Y
	11	0.069	NA	0.019	0.000	0.211	0.023			Y
	15	**0.140**	0.035	0.043	0.000	0.194	0.042			Y
QDihex2	10	0.046	0.024	0.050	0.001	0.417	0.068	0.837	0.171	N
	11	0.032	NA	0.030	0.000	0.377	0.046			N
	15	0.047	0.024	0.055	0.003	0.380	0.062			N
EllagicPent1	10	0.162	0.215	0.171	0.047	0.360	0.058	0.798	0.016	N
	11	0.063	NA	0.069	0.018	0.157	0.026			N
	15	0.194	0.242	0.243	0.075	0.568	0.095			N
EllagicPent2	10	0.073	0.031	0.048	0.000	0.160	0.040	0.843	0.107	Y
	11	0.026	NA	0.022	0.002	0.075	0.016			Y
	15	0.120	0.044	0.095	0.009	0.413	0.079			Y
Lambertianin	10	2.699	3.578	2.457	0.847	4.286	0.629	0.648	0.071	N
	11	0.578	NA	0.579	0.151	1.315	0.222			N
	15	0.917	1.223	1.086	0.419	2.111	0.321			N
Ellagic	10	0.057	0.040	0.044	0.009	0.155	0.021	0.446	0.000	N
	11	0.013	NA	0.011	0.001	0.101	0.008			N
	15	0.391	0.284	0.308	0.039	1.505	0.219			N
SanguiinH2	10	0.036	0.034	0.029	0.012	0.058	0.008	0.544	0.130	N
	11	0.017	NA	0.015	0.003	0.033	0.006			N
	15	0.018	0.014	0.015	0.007	0.048	0.005			N
SanguiinH6	10	5.496	5.965	5.020	2.147	7.419	0.914	0.630	0.085	N
	11	1.297	NA	1.323	0.604	2.889	0.391			N
	15	2.918	2.992	2.977	1.695	4.682	0.598			N
QHexPent	10	0.008	0.075	0.037	0.000	0.193	0.041	0.864	0.250	Y
	11	0.005	NA	0.028	0.000	0.134	0.031			Y
	15	0.014	0.078	0.064	0.001	0.245	0.060			N
QHexRham1	10	0.074	0.035	0.021	0.000	0.147	0.031	0.802	0.278	Y
	11	0.027	NA	0.012	0.000	0.077	0.017			Y
	15	0.041	0.044	0.023	0.000	0.156	0.032			N
QGal	10	0.232	0.086	0.072	0.001	0.376	0.069	0.838	0.182	Y
	11	0.106	NA	0.053	0.000	0.263	0.052			Y
	15	0.142	0.109	0.104	0.000	0.450	0.096			N
**QGlcU**	10	**1.356**	0.431	0.457	0.038	1.186	0.244	0.417	0.128	N
	11	0.506	NA	0.280	0.015	1.042	0.151			N
	15	**0.633**	0.345	0.516	0.096	1.356	0.204			N
QHexRham2	10	0.067	0.036	0.034	0.000	0.145	0.031	0.894	0.148	Y
	11	0.031	NA	0.015	0.000	0.105	0.016			Y
	15	0.038	0.039	0.023	0.000	0.178	0.027			Y
QGlu	10	0.124	0.097	0.062	0.011	0.255	0.035	0.590	0.101	N
	11	0.058	NA	0.045	0.002	0.164	0.025			N
	15	0.057	0.060	0.077	0.015	0.221	0.035			N
**QPent**	10	0.009	**0.134**	0.064	0.000	0.315	0.076	0.857	0.219	Y
	11	0.002	NA	0.046	0.000	0.261	0.051			Y
	15	0.013	**0.095**	0.107	0.001	0.431	0.101			Y
KGal	10	0.029	0.007	0.006	0.000	0.050	0.008	0.846	0.185	Y
	11	0.010	NA	0.005	0.000	0.050	0.006			Y
	15	0.014	0.010	0.009	0.000	0.062	0.010			Y
QMalGlu	10	0.038	0.033	0.018	0.001	0.087	0.011	0.616	0.051	N
	11	0.018	NA	0.014	0.000	0.046	0.009			N
	15	0.013	0.018	0.018	0.001	0.061	0.012			N
MeEllPent	10	0.055	0.074	0.062	0.015	0.169	0.029	0.817	0.000	N
	11	0.018	NA	0.022	0.004	0.069	0.011			N
	15	0.079	0.082	0.086	0.018	0.251	0.041			N
EllAcetylPent	10	0.087	0.123	0.072	0.003	0.235	0.048	0.815	0.214	N
	11	0.027	NA	0.021	0.000	0.092	0.017			N
	15	0.080	0.116	0.078	0.002	0.284	0.051			N
**KGlcU**	10	**0.210**	0.038	0.044	0.001	0.204	0.034	0.367	0.077	N
	11	0.068	NA	0.028	0.000	0.169	0.021			N
	15	**0.073**	0.032	0.047	0.003	0.177	0.030			N
KGlu	10	0.043	0.027	0.008	0.000	0.080	0.013	0.311	0.472	Y
	11	0.014	NA	0.010	0.000	0.045	0.008			N
	15	0.016	0.016	0.020	0.001	0.071	0.012			N
QHex	10	0.025	0.010	0.012	0.001	0.038	0.008	0.763	0.092	N
	11	0.010	NA	0.005	0.000	0.022	0.004			N
	15	0.013	0.007	0.011	0.002	0.045	0.007			N

10, 11 and 15 represent samples from years 2010, 2011 and 2015

TA = total anthocyanin content by the colorimetric method, values in mg cyanidin glucoside equivalents/100 g fresh weight. Metabolites are denoted by their abbreviated names, see Table 1. NA = value not available due to no Moy samples that year. Mean, Min, Max, SD = mean, minimum, maximum and standard deviation of the offspring

H_G = generalised heritability of the genotype (log_10_ scale); H_GY = generalised heritability of the genotype × year effect (log_10_ scale). BM = Y if the metabolite distribution was clearly bimodal, N otherwise. Metabolites in bold were significantly higher in that parent (p < 0.05) in that year

Histograms of the distributions of the log-transformed metabolites showed several metabolites had bimodal distributions, as recorded in Table 2. This was usually consistent across all three years, although for some there was no clear bimodality in 2015. As discussed below, these distributions are associated with major QTLs detected on linkage groups (LG) 1, 4 and 6. The generalised heritability for the genotypes varied from 0.31 to 0.89 (Table 2). The highest heritabilities were found mainly for the metabolites with bimodal distributions: the anthocyanins PelRut, PelGluRut, CySophRham/CyGluRut, CyXylRut/CySamRham, CyC_34_H_40_O_21_, the flavonols KGal, QGal, QPent, QHexPent, QDihex1, QDihex2, QGluRut1, QGluRut2, QHexRham1, QHexRham2 and the ellagic acid derivative EllagicPent2. The lowest generalised heritabilities were found for KGlu, KGlcU, QGlcU and Ellagic, with values of 0.31–0.45. The generalised heritability of the genotype x year effect was much lower, varying from 0.00 to 0.28 apart from for KGlu, estimated as 0.47.

Because of the bimodal distributions for several metabolites, a simple correlation coefficient does not represent the relationship among the metabolites well in some cases. Supplementary Figures S2A, S2B and S2C show scatter plot matrices for the anthocyanins, flavonols and ellagitannins/ellagic acid derivatives respectively, based on the log-transformed genotype means for 2010 and Figure S2D shows relationships between the flavonols and anthocyanins. The figures for the other years (not shown) are similar. As an example, consider the correlation between CySoph and CyXylRut/CySamRham in Fig. S2A. The Pearson correlation coefficient was − 0.50 but the scatter plot showed that some genotypes had very low levels of CyXylRut/CySamRham, and the other genotypes formed two clusters, within each of which the correlation with CySoph was positive. Another interesting pair were CySoph and CySam. The individual distributions for these metabolites were unimodal but the scatter plot showed two clear clusters. Likewise, MeEllPent and EllAcetylPent in Fig. S2C showed clear clustering and a positive correlation within the clusters, but unimodal individual distributions and a negative correlation overall. These plots will be discussed in more detail in the QTL results below.

### QTL mapping

**Fig. 1:**
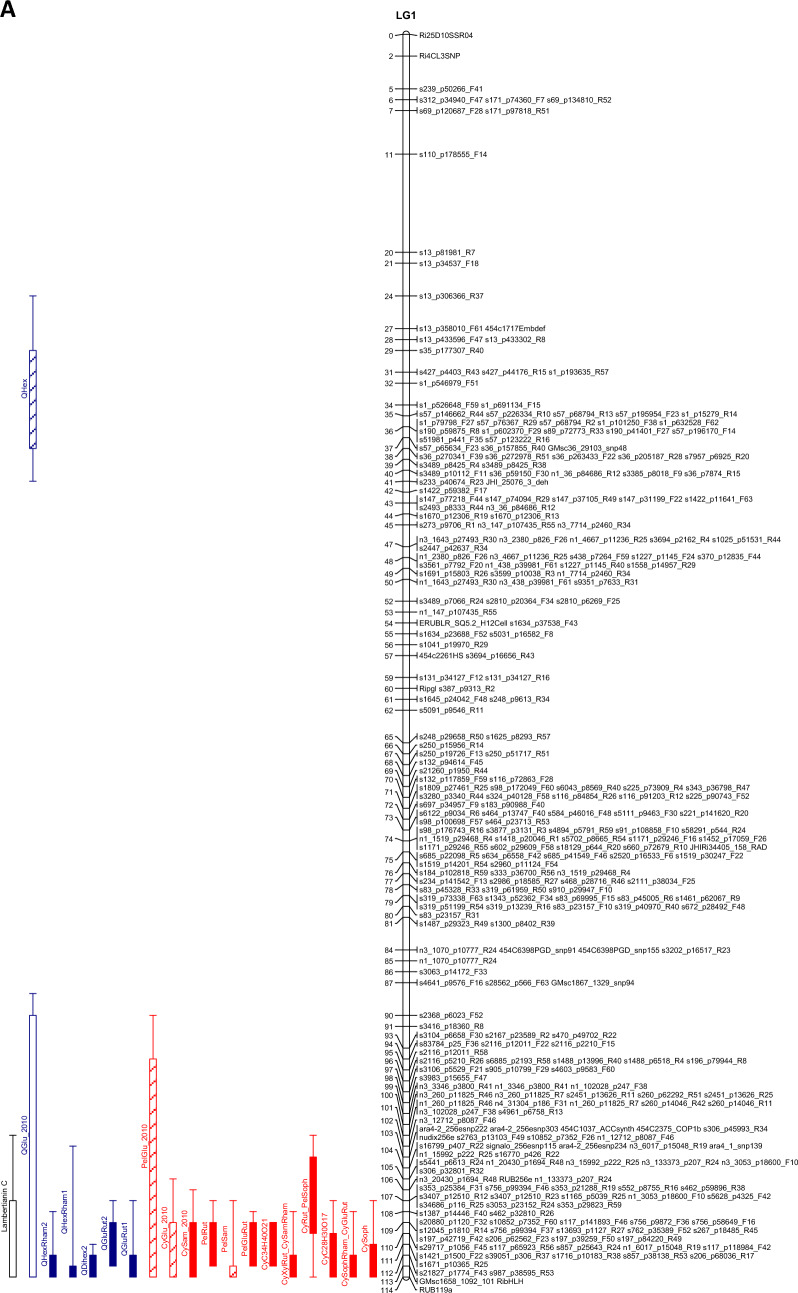

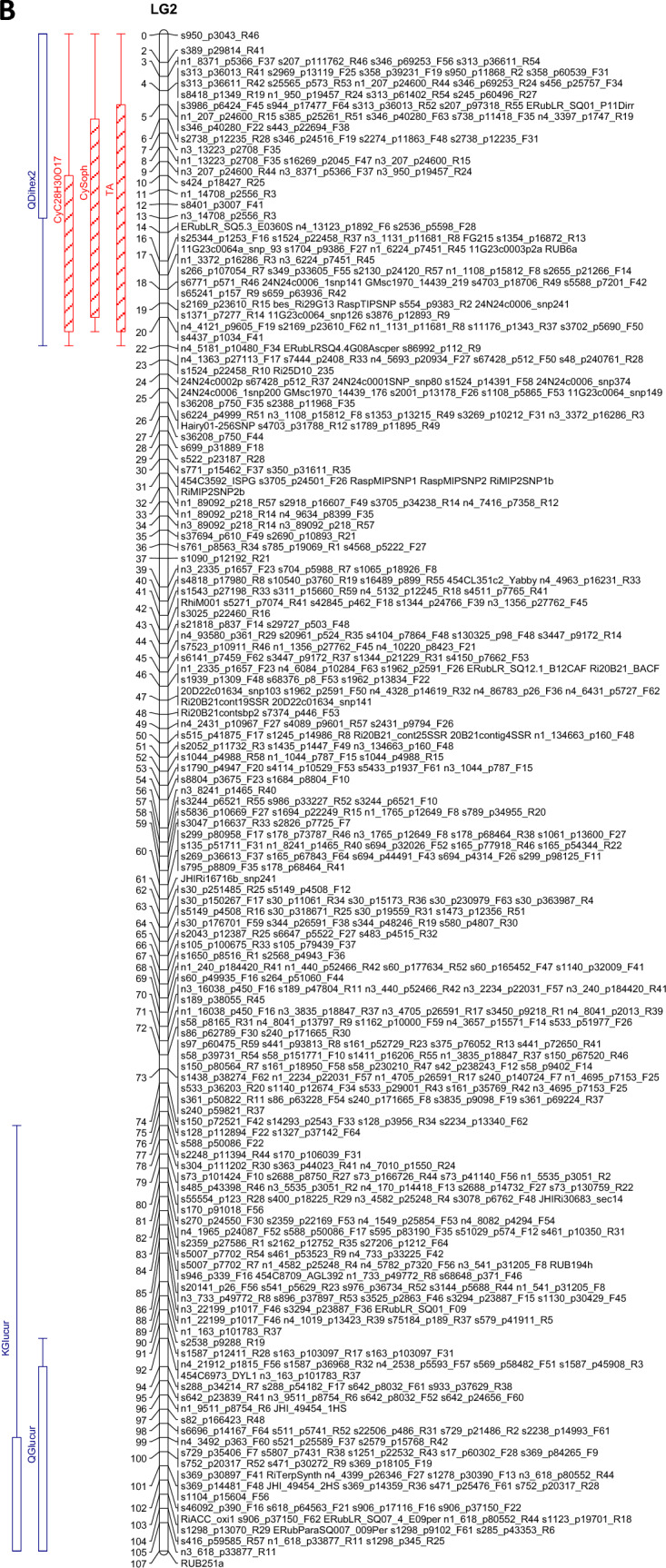

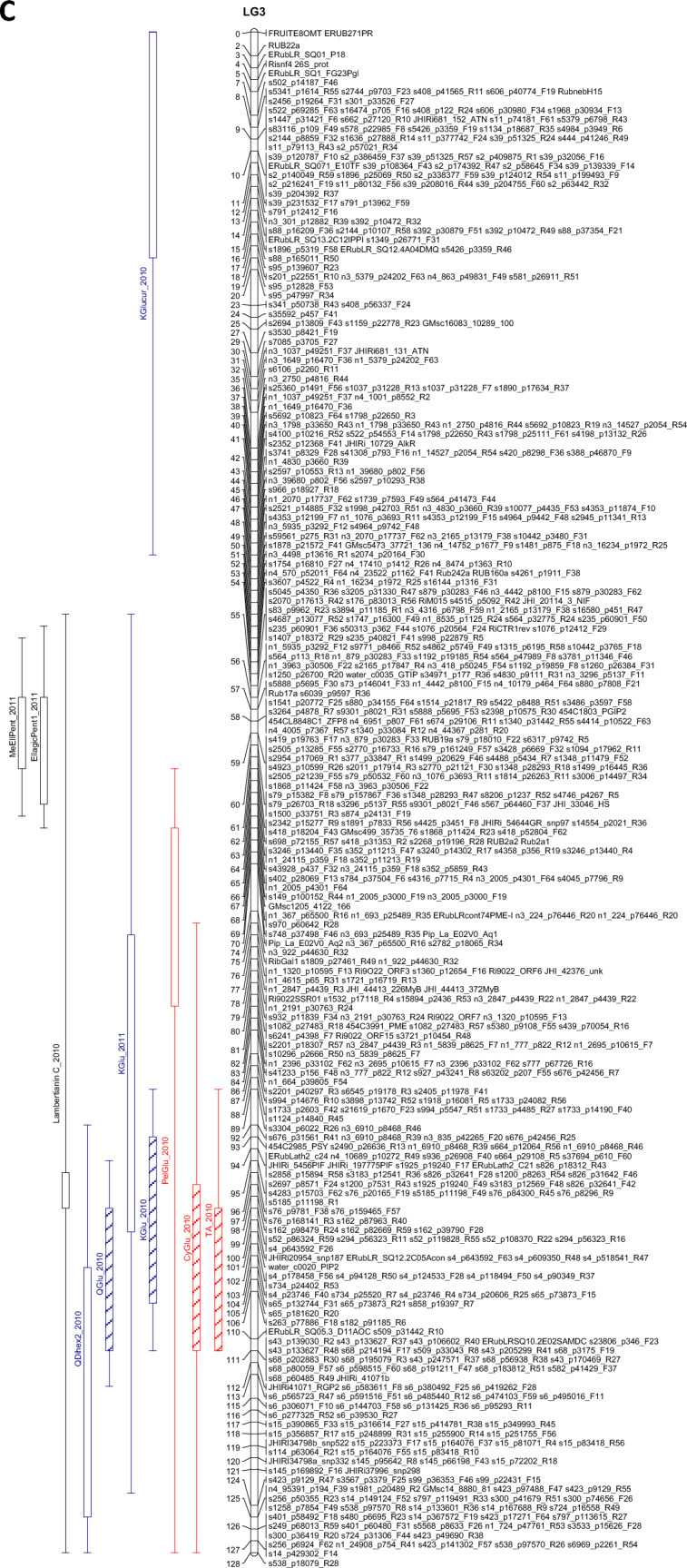

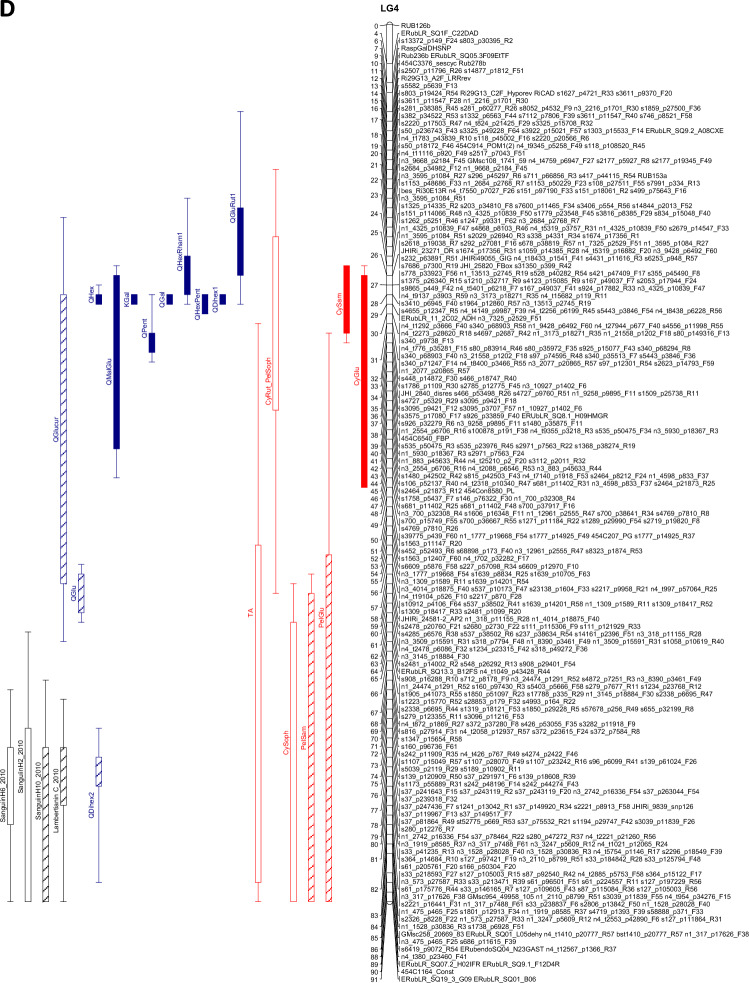

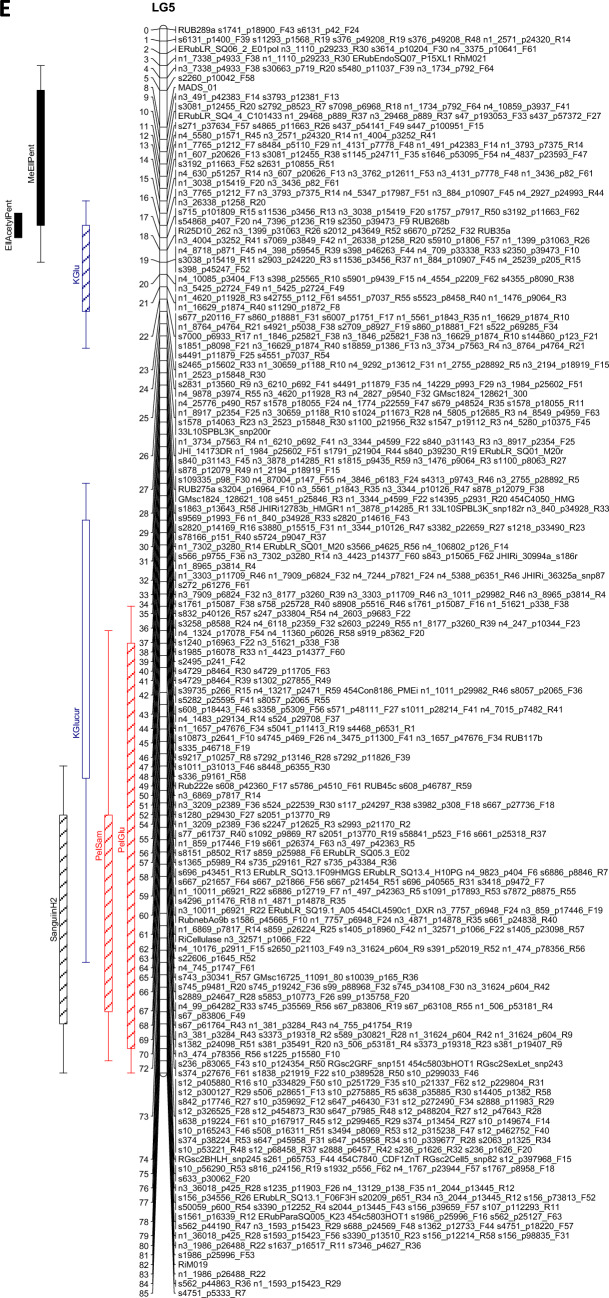

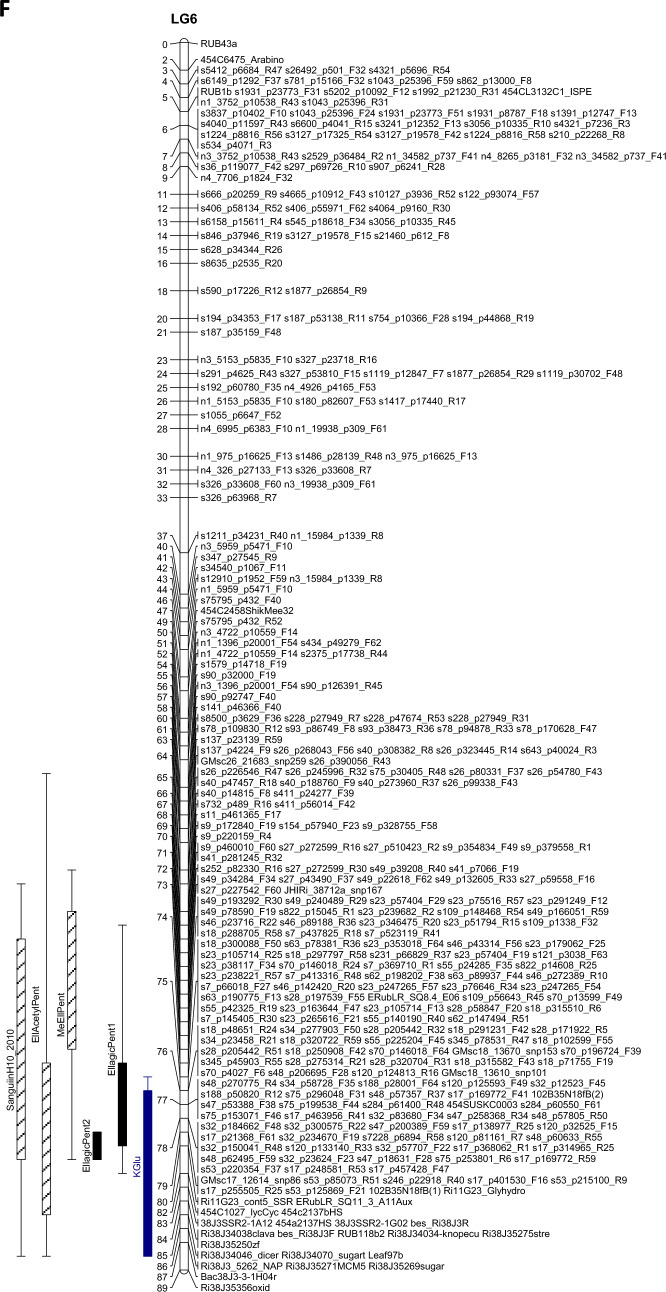
Linkage maps showing QTL locations. The boxes show the one-LOD support interval, and the whiskers show the two-LOD interval. Major QTLs are shown as solid boxes, strong QTLs as diagonally filled boxes and minor QTLs as unfilled boxes. Red = anthocyanins, blue = flavonols, black = other traits. **A**–**F** show linkage groups (LG) 1–6

The QTL analysis generally showed a good consistency over years, but there were also some significant QTL × year interactions. These were however generally associated with different sizes of QTL effects, rather than a different direction of the effect. QTLs were detected in each year on raspberry LG1-LG6, but not on LG7 (Table 3). This is in accordance with previous studies on this population (e.g. Hackett et al., 2018), which have seldom found QTLs on LG7. Figure 1 shows linkage maps with one- and two-LOD support intervals for the QTLs. The term ‘major’ QTL is used to refer to a QTL with LOD greater than 10, ‘strong’ QTL for a QTL with LOD between 5 and 10 and ‘minor’ QTL for a QTL with LOD above the permutation threshold of 3.8 but below 5. The results presented below are from the over-year averages unless otherwise stated. Linkage groups LG1, LG4 and LG6, with QTLs large enough to give bimodal distributions for some metabolites, are discussed first, followed by the groups with smaller QTLs.

**Table 3:**
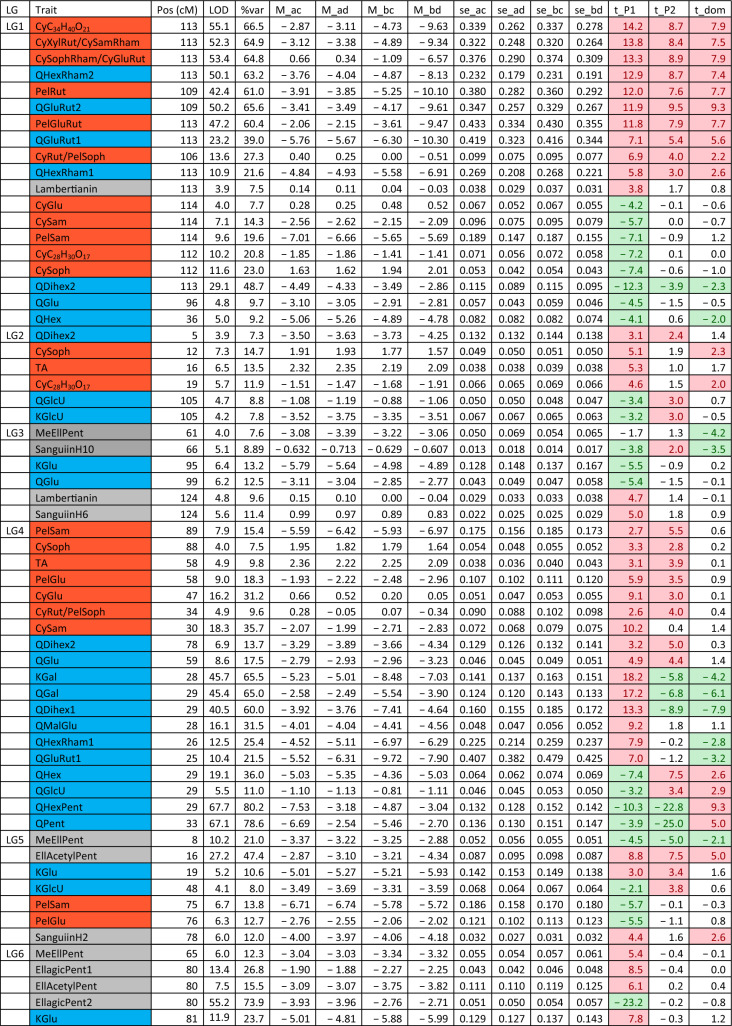
QTLs detected for each metabolite. This is based on the average values for each genotype over the three years

Pos is the position of the maximum LOD, %var is the %variance explained by that QTL. M_ac, M_ad, M_bc and M_bd are the estimated means for offspring genotypes ac, ad, bc and bd respectively and se_ac etc.are the corresponding standard errors. t_P1 is a t-test for the significance of the Latham additive effect, t_P2 is a t-test for the Glen Moy additive effect and t_dom for the dominance effect. The significant effects are shaded red if they are positive or green if they are negative. Anthocyanin components are shaded in red, flavonols in blue, with ellagitannins and ellagic acid derivatives in grey

#### LG1

**Fig. 2:**
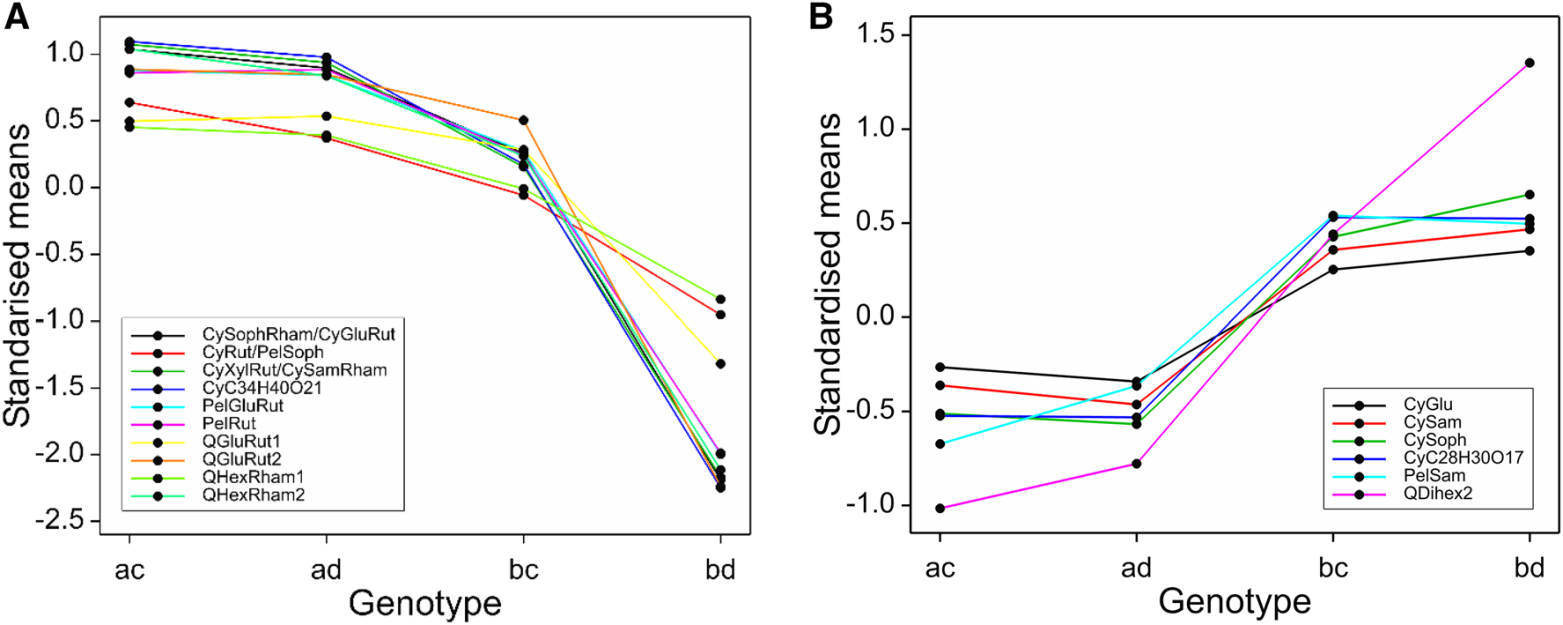
**A** Genotype means from the over-years analysis (on log_10_ transformed data) for metabolites at the QTLs at the bottom of LG1. The genotype bd results in lower levels of these metabolites than the other genotypes. Each genotype mean is standardised by subtracting the overall mean and then dividing by √(QTL residual mean square) for comparability. **B** Genotype means from the over-years analysis (on log_10_ transformed data) for metabolites at the QTL at the bottom of LG1. The ac and ad genotypes have lower levels of the components, an effect mainly due the Latham parent. Each genotype mean is standardised by subtracting the overall mean and then dividing by √(QTL residual mean square) for comparability

There was a very significant region at the bottom of LG1 for all three years, with many metabolites mapping to positions from 106 to 113 cM, with LODs up to 55.1 and the % variance explained (R^2^) by this QTL up to 67%. This is close to the RibHLH marker at 113 cM, which was identified by McCallum et al. (2010) as close to a QTL for a smaller set of anthocyanins than measured in this study. One group of metabolites had major QTLs (explaining between 22 and 67% of the trait variance) and showed a pattern where the levels were close to zero for one genotype (bd), higher for bc and highest for ac and ad (Fig. 2A). This pattern was shown by the anthocyanins, CyC_34_H_40_O_21_, CySophRham/CyGluRut, CyXylRut/CySamRham, PelGluRut, PelRut and CyRut/PelSoph and the flavonols, QHexRham2, QGluRut2, QGluRut1 and QHexRham1. All these flavonoid components contain attached rhamnose groups and even the unidentified cyanidin derivative CyC_34_H_40_O_21_ gave a neutral loss of 146 under MS fragmentation which is consistent with the presence of a rhamnose group (Table 1). The presence of the major QTLs is responsible for the bimodal distribution shown by all these metabolites apart from CyRut/ PelSoph. The genotype bd, with the lowest values, represents a clear separate cluster in the scatter plot matrices (see supplementary Figs S2A, S2B, & S2D). The metabolites are all positively correlated, both for the overall correlation and for the correlation within the genotypes ac, ad and bc, which have higher values of each metabolite than bd (Fig. 2A).

A further set of metabolites mapped to the same location (109–114 cM) on LG1, but with smaller QTLs, and gave a different pattern of segregation and more variation over the years (Fig. 2B). All these components are flavonoid glucosides with glucose groups directly attached to the flavonoid aglycone (e.g. CyGlu) or with further glycosyl groups attached to the flavonoid glucosyl core (e.g. cyanidin 3-sambubioside; CySam or cyanidin sophoroside; CySoph). A major QTL for the flavonol QDihex2 was also detected in this region for all three years. Major QTLs were detected in 2010, with smaller QTLs that are consistent in position and sign in 2011 and 2015, for the anthocyanins CySoph, CyC_28_H_30_O_17_, and PelSam. A major QTL for CySam and strong QTLs for CyGlu and PelGlu were also detected in this region in 2010 only, together with a minor QTL nearby, at 96 cM, for QGlu in 2010 only. For all these metabolites, the difference in the genotype means is due to the allele received from Latham, with lower values for the ‘a’ allele (in contrast to the flavonoids shown in Fig. 2A), where the ‘a’ allele had high levels.

All the anthocyanins that map here showed positive correlations (Fig S2A), particularly PelGlu and PelSam, with a correlation of 0.90 in 2010, and CyGlu and CySam, with a correlation of 0.88. The correlations were also high in 2011 and 2015. These correlations may be expected as the glucosides could be metabolic precursors of the sambubioside derivatives, which have xylose groups added at position 2 of the glucose moiety which is attached to the anthocyanidin (Bradish et al., 2012). In addition, there was a strong correlation between PelGluRut and PelRut (0.95) which also could reflect a -precursor relationship (Fig. S2A). The strong correlation (0.93) between the potential cyanidin derivative CYC_34_H_44_O_21_ and CyXylRut could also suggest a precursor-intermediate relationship. However, the strong correlation (0.95) between PelRut and CyXylRut is not so readily explained.

For some pairs of metabolites, the overall correlations do not give a satisfactory representation of the true relationships between these anthocyanins and those with the alternative pattern above. For example, the overall correlation between CyXylRut/CySamRham and CySoph was − 0.50 in 2010 (Fig. S2A). However, apart from the cluster of values from genotype bd, which has very low levels of CyXylRut/CySamRham (see red arrow in Fig. S2A), the relationship within the other three clusters is positive. Indeed, CySam and CyXylRut/CySamRham (overall r = − 0.18 in 2010) and CyGlu and CyXylRut/CySamRham (overall r = − 0.10 in 2010) showed the same pattern with low levels of CyXylRut/CySamRham due to the genotype bd.

Supplementary Figure S2D shows the relationships between anthocyanins and flavonols. There were many near-zero correlations, but some components showed high positive correlations. For example, CySophRham/CyGluRut had strong correlations with QGluRut2 (r = 0.97), QHexRham2 (0.97) and QGluRut1 (0.72). A similar pattern of correlation was seen between PelGluRut with QGluRut2 (0.90), QHexRham (0.89) and QGluRut1 (0.61) and between PelRut with QGluRut2 (0.91), QHexRham (0.93) and QGluRut1 (0.71). In all these cases, the lower correlation with QGluRut1 was influenced by low levels associated with one genotype. The biochemical feature that links these components is the addition of similar glycosyl groups to the flavonol or anthocyanidin core, so it is intriguing to suggest that these steps are under common genetic control. In addition, QDihex2 also had a correlation of 0.82 with CySoph, again suggesting a commonality of added glycosyl groups.

There were some candidate genes underlying this QTL region (LG1, 106–114 cM) that could be involved in the control of anthocyanin and flavonol biosynthesis. Firstly, there was a homologue (> 80% homology with genes from *Rosa chinensis*) of a leucine-rich repeat protein kinase that has been associated with the accumulation of polyphenols in Tannat grapes (Da Silva et al., 2013). There was a homologue of a MYB39 transcription factor (> 80% homology to genes from *R. chinensis* and *Fragaria vesca*) that has been associated with microRNA control of phenylpropanoid biosynthesis in *Arabidopsis* (AT3G61250; Sharma et al., 2016) and another MYB3-like transcription factor (homology to AT1G22640.1; ≥ 85% homology with *R. chinensis* and *F. vesca* genes) that was upregulated in polyphenol-rich blueberry skins vs flesh (Plunkett et al., 2018). There was a homologue of a C2H2-type zinc finger protein (> 75% homology to *R. chinensis* and *F. vesca* genes), which was found to be upregulated in ripe versus unripe grapes (Ahn et al., 2019). Also, there was a homologue to a structural biosynthetic gene that could be directly involved in the formation of the anthocyanidins, i.e. an anthocyanidin reductase (AT1G61720.1; ANR; > 80% homology with Prunus genes), which was upregulated in the polyphenol-rich skin of blueberries versus blueberry flesh (Plunkett et al., 2018) and implicated in polyphenol accumulation in grapes (Bogs et al., 2005).

Also, there was a homologue to an Arabidopsis gene (~ 80% homology to *R. chinensis and F. vesca* genes) that encoded a UDP-glycosyl transferase (UDPGT) that has been implicated in polyphenol biosynthesis in ripe grape berries (Ahn et al., 2019). This could be responsible for the attachment of glycosyl groups directly to the flavonoid aglycone (e.g. to the cyanidin group in CyGlu) and/or in adding extra glycosyl groups to the flavonoid glucoside (as discussed for components in Fig. 2B).

Despite the QTLs for individual anthocyanins, no QTL was detected for Total Anthocyanin content (TA) on LG1. This appears to be because the increase in the levels of some of the individual anthocyanins is balanced by the decrease in the levels of others. This was also the case in a previous study (McCallum et al., 2010) which examined levels of eight major raspberry anthocyanins. A strong QTL for QHex was also detected at 36 cM on LG1. This was an effect mainly of the Latham parent.

#### LG4

**Fig. 3:**
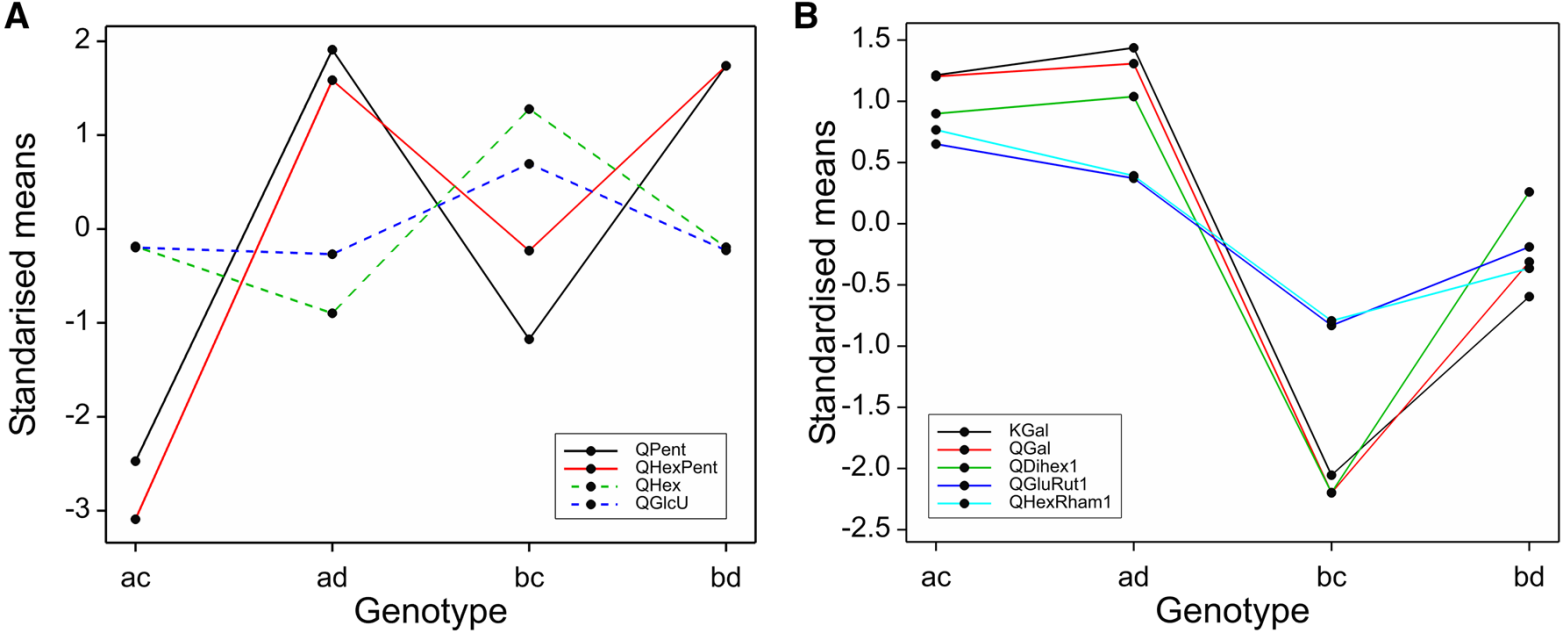
**A** Genotype means from the over-years analysis (on log_10_ transformed data) for the QTL at 25–33 cM on LG4 (and the metabolites where the QTL effects are different from Fig. **B**). Each genotype mean is standardised by subtracting the overall mean and then dividing by √(QTL residual mean square) for comparability. **B **Genotype means from the over-years analysis (on log_10_ transformed data) for the QTL at 25–33 cM on LG4. Each genotype mean is standardised by subtracting the overall mean and then dividing by √(QTL residual mean square) for comparability. The levels of the metabolites were lowest in genotype bc

There was a very significant area on LG4 from 25 to 33 cM for all three years, especially for the flavonol metabolites, with QTLs with LODS up to 67.7 and R^2^ up to 80%. This is close to the region detected by Kassim et al. (2009) and McCallum et al. (2010). Markers ERubLR_SQ8.1_H09HMGR, JHIRi49055_GIG and ERubLR_11_2C02_ADH are in this region, also marker RiCAD is nearby. The flavonol metabolites mapping to this region showed three distinct genotype patterns. The most significant metabolites were QHexPent and QPent, which had major QTLs in this region in all three years (with LODs of 67.1 to 67.7 and R^2^ from 78.6 to 80.2%) with associated bimodal distributions. For these metabolites genotype ac had the lowest level, then bc, with ad and bd highest (Fig. 3A). They are also very highly correlated (r = 0.90 in 2010; Fig. S2B) which could indicate a precursor- product relationship. QHex also had a major QTL here but the genotype pattern was different, with ad having the lowest level, ac and bd having intermediate levels and bc having the highest level. QGlcU also had a strong QTL in this region with the same genotype pattern as QHex.

The relationship between the pentose containing flavonols and the genotypes could be explained if this QTL overlaid genes involved in sugar addition to the flavonols. However, the metabolic relationship between QHex and QGlcU is less obvious.

Other flavonols KGal, QGal and QDihex1 also had major QTLs in this region, with LODs of 40.5 to 45.7 and R^2^ from 60.0 to 65.5%. For these, the lowest levels were for genotype bc, higher for bd and the highest for ac and ad (Fig. 3B). This region was associated with the observed bimodal distribution for these metabolites. KGal, QGal and QDihex1 were positively correlated, with correlations of at least 0.88 in 2010 (Fig S2B), and similarly high for the other years. QHexRham1 and QGluRut1 also had major QTLs here (LODs of 10.4–12.5) with a similar genotype pattern. QMalGlu also mapped with a major QTL here, with a similar pattern but less distinction between bc and bd. It is intriguing that these flavonol components share attached hexosyl groups (i.e. either glucosyl or galactosyl) and perhaps this region controls the addition of such sugar groups.

**Fig. 4:**
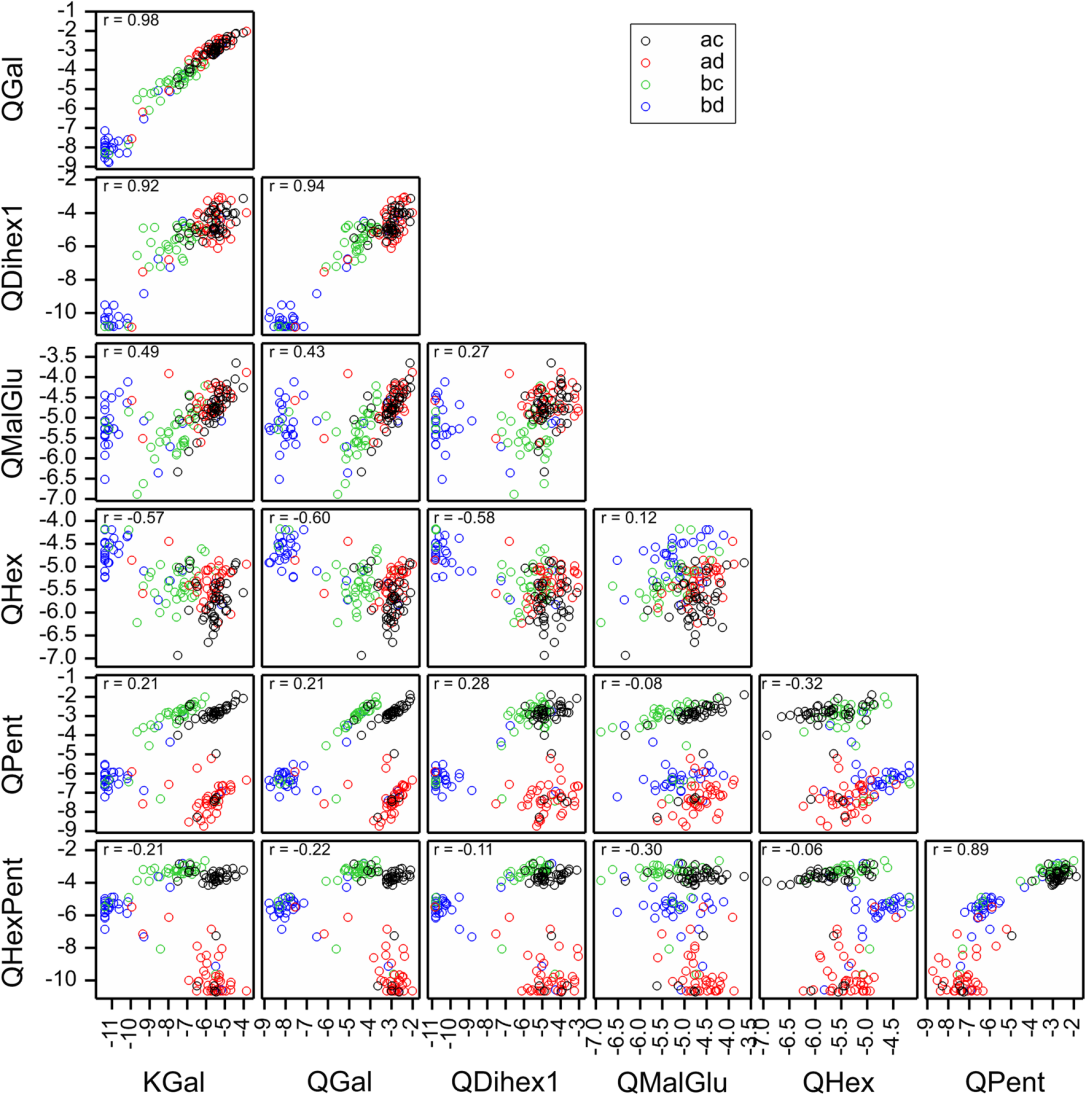
Scatterplot matrix of flavonols with major QTLs on LG4, labelled by the genotypes at 30 cM (based on SNPs s340_p9738_F13 from Latham and n1_9428_p6492_F60 from Glen Moy). The colour coding reflects the alleles inherited from the parents, ac, ad, bc & bd

Scatterplots of these flavonols show a strong structure with up to four groups corresponding closely to the genotypes at about 30 cM on LG4 (Fig. 4). This is generally backed up by the scatter plots for flavonols shown in Fig. S2B. The ac genotype (black) and ad genotype (red), which inherit the “a” allele from Latham, both had high levels of KGal, QGal, QDihex1, QMalGlu and QHex but the ac genotype had much higher levels than the ad genotype for QPent and QHexPent. The ac genotype and bc genotype (green) inheriting allele c from Glen Moy both had high QPent and QHexPent but the bc genotype (green) had lower levels of KGal, QGal, QDihex1 and QMalGlu. There were strong correlations (~ 0.9) between the levels of QGal and QDihex1 across the progeny in 2010, which could reflect precursor relationships (Fig. 4 & Fig. S2). This fits with the addition of a second hexose group to QGal. A similar precursor-product relationship may exist between the kaempferol and quercetin hexosides 1 (probably Gal), which differ by one hydroxyl group on the A-ring. Indeed, a similar strong correlation (r = 0.92) was noted between KGlcU and QGlcU (Fig. S2c). This could suggest metabolic interconversion of kaempferol to quercetin glycosides.

Strong QTLs were also detected for QGlu and QDihex, but these were well separated from the above, with peak LODs at 59 cM and 78 cM respectively. Both had highest levels for genotype ac, intermediate for ad and bc and lowest for bd.

Some QTLs for anthocyanins were also detected on LG4, with good consistency over years, but the QTL positions for each anthocyanin varied more. A major QTL for CySam mapped to 30 cM with LOD 18.3, and R^2^ of 35.7%. This was mainly an effect of the Latham parent. Another major QTL for CyGlu was mapped to 47 cM, with LOD 16.2, and R^2^ of 31.2%, and a strong QTL for PelGlu and a minor QTL for Total Anthocyanin mapped to 58 cM. All these showed a similar pattern of genotype means, with ac having the highest level, ad and bc having intermediate levels and bd having the lowest level. PelSam had a strong QTL at 89 cM and CySoph had a minor QTL at 88 cM, with similar patterns to the previous three anthocyanins.

An interesting pattern was noted in the scatterplots for the data in 2010 (Fig. S2a, top left box) for CySoph and CySam. CySam is a disaccharide consisting of xylose and glucose attached to cyanidin whereas CySoph is cyanidin with an attached disaccharide of glucose. These showed two distinct parallel clusters related to the genotypes. The marker that corresponds best to the separation is JHIRi49055_GIG at 26 cM on LG4, which segregates in Latham only: the clusters correspond closely to the offspring receiving the a and b alleles from Latham. Similar patterns were observed in the other years (not shown).

**Scheme 1:**
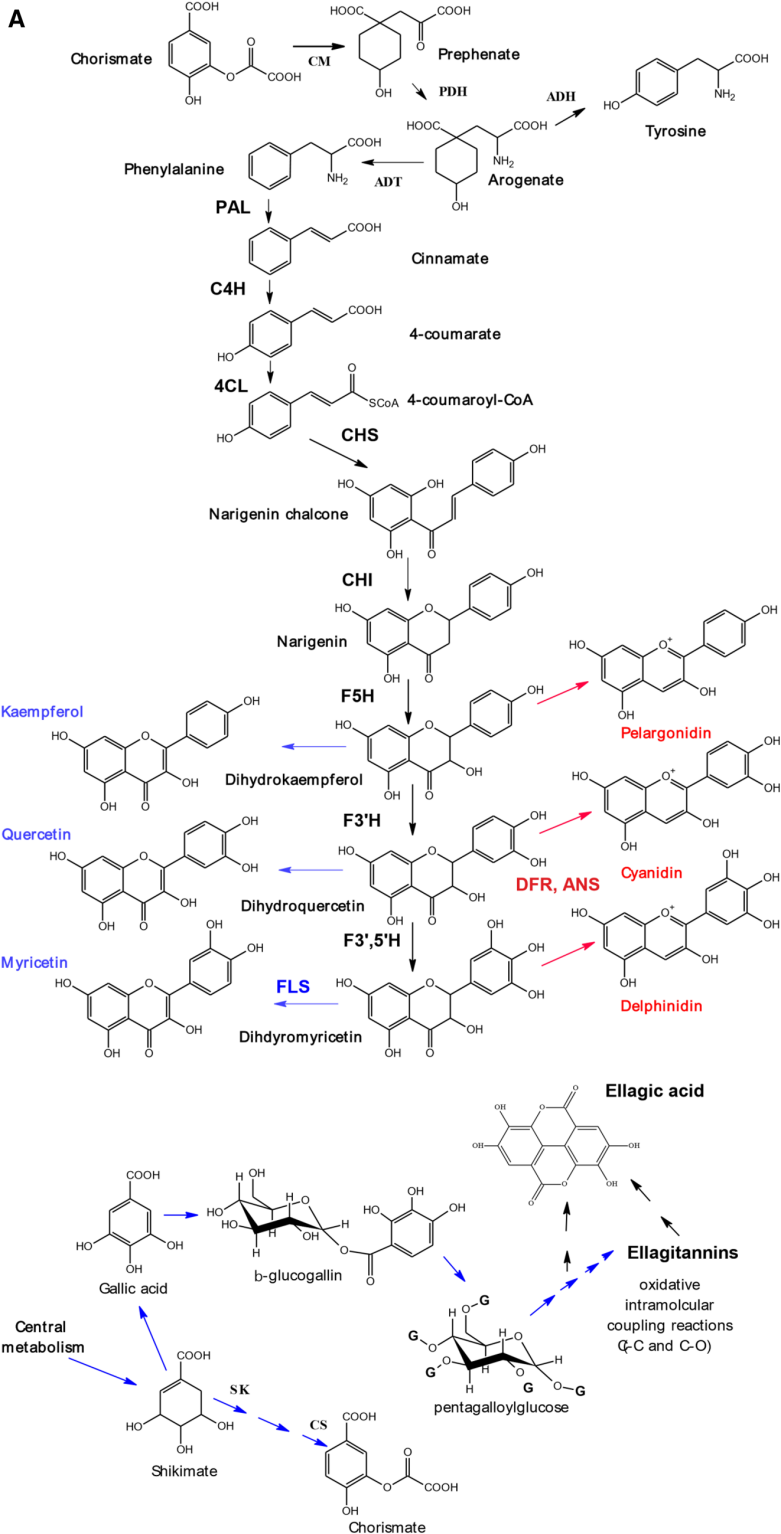

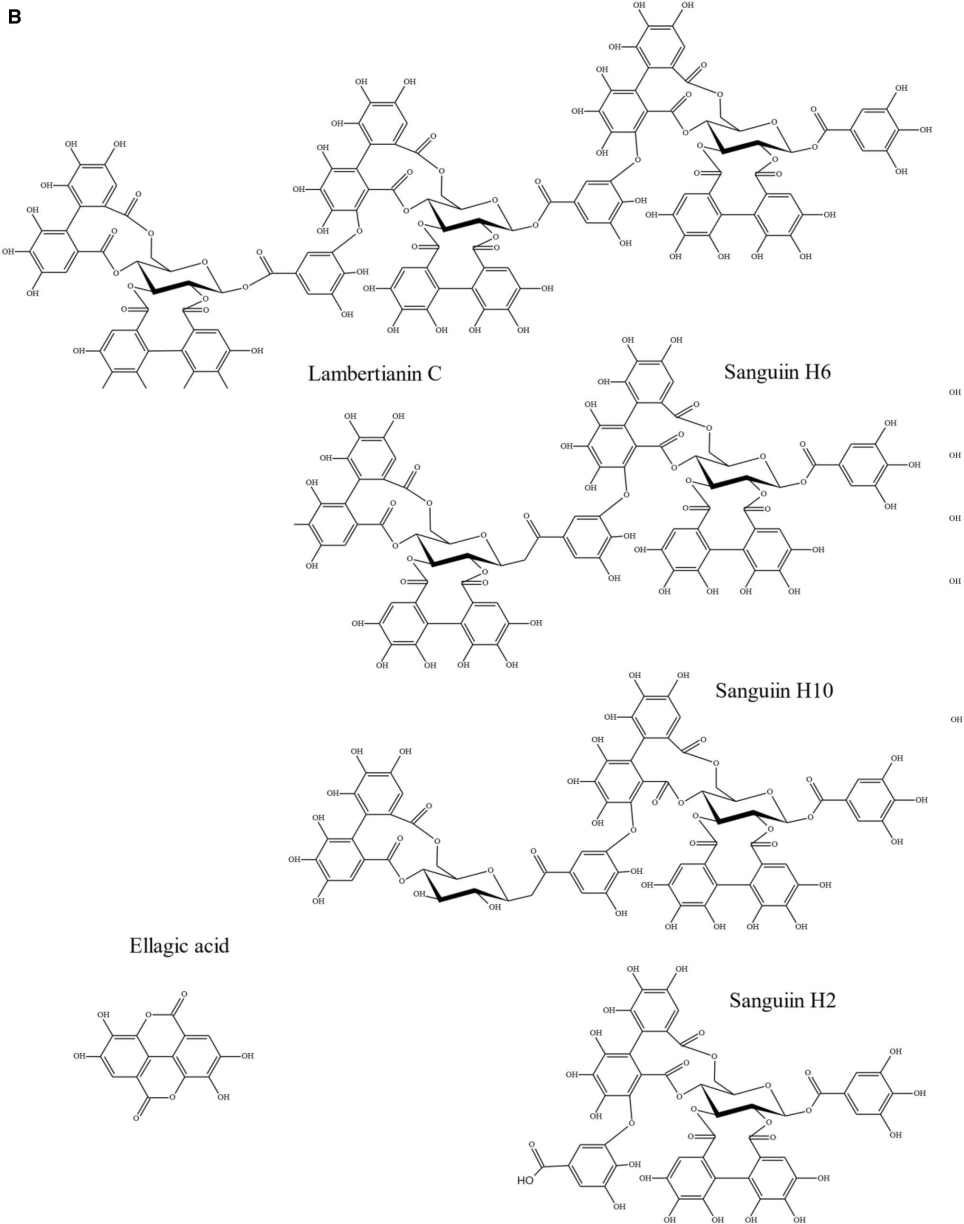
**A** Pathway for anthocyanin and flavonol biosynthesis. *Insert of possible biosynthetic pathway to ellagitannins and ellagic acid. *Enzymes are abbreviated as follows: ADH, arogenate dehydrogenase; ADT, arogenate dehydratase; ANS, anthocyanin synthase; CHI, chalcone isomerase; CHS, chalcone synthase; C4H, cinnamate-4-hydroxylase; 4CL, 4-coumarate ligase; CM, chorismate mutase; DFR, dihydroflavonol reductase; F3H, flavonone-3-hydroxylase; F5H, ferulate 5-hydroxylase; F3′H, flavonoid-3′-hydroxylase; F3′5′H, flavonoid-3′,5′-hydroxylase; FLS, flavonol synthase; PAL, phenylalanine ammonia lyase; PDH, prephenate dehydratase; SK = Shikimate kinase and CS = chorismate synthase. For clarity, only enzymes that produce the flavonoid aglycones are shown, not the glycosyltransferases and acyl transferases that decorate the aglycones. Methylated anthocyanidins (i.e. peonidin, petunidin and malvidin) and flavonols (isorhamnetin) are also not shown as they are not present in raspberry. **B **Structures of ellagitannins and ellagic acid found in raspberry

There were some genes underlying the QTL region at 25–33 cM on LG4 that have been implicated in the control of polyphenol biosynthesis. There was a homologue of a cytochrome P450 that has been implicated in anthocyanin accumulation in purple Brassicas (Zhu et al, 2021). Also, there was a gene with homology to a dihydroflavonol 4-reductase (DFR)-like protein in *Arabidopsis* (Yuan et al., 2007) (also 85% homology with the *R. chinensis* gene) which could be involved in the final steps of anthocyanidin biosynthesis (see scheme 1A).

However, most interestingly there were homologues of genes encoding uridine diphosphate glycosyltransferases (UDPGTs) which have been implicated in the glycosylation of flavonoids, and in particular anthocyanins. All the homologies were > 80% with the genes from *R. chinensis* or *F. vesca*. Indeed, one hit was noted with UDPGT 74E2 (AT1G05680.1) which has been described as an anthocyanin 3-*O*-glucoside: 2″-O-xylosyl-transferase that converts cyanidin 3-*O*-glucoside to cyanidin 3-*O*-xylosyl-glucoside in *Arabidopsis* [AT5G54060(UF3GT) (arabidopsis.org)]. This is interesting as CySam (or cyanidin 3-*O*-xylosyl-glucoside) has a major QTL in this region. Also, the transfer of xylose would also be relevant to the formation of the flavonol pentoses (QPent and QHexPent), which also have major QTLs here. It would be intriguing if this UDPGT homologue could also be involved with the transferal of hexosyl units to form the other anthocyanidin and flavonol hexose components associated with this QTL region.

In 2010 at 77 cM on LG4, there was a strong QTL for the ellagitannins Lambertianin C and Sanguiin H10 and minor QTLs for Sanguiin H6, and Sanguiin H2. These had minor QTLs in a similar region in 2011 (77–89 cM) and there was a minor QTL for Lambertianin C at 84 cM in 2015 only. However, no significant QTLs were detected here for the over-years means. These metabolites are all positively correlated from 0.52 up to a maximum of 0.86 for the correlation between Lambertianin C and Sanguiin H6 in 2010 (Fig. S2c). Correlations for other years were similar (results not shown). Ellagitannins (ETs) originate from gallic acid, itself formed from the central metabolite, shikimate (Scheme 1A). The production of galloyl glucose is the first committed step in the formation of Ets (e.g. Schulenburg et al., 2016) then further galloyl groups are added to form pentagalloyl glucose. Ets are then produced by defined intramolecular oxidative coupling reactions on pentagalloyl glucose with a C–C coupling of galloyl groups producing the hexahydroxydiphenic (HHDP) acid unit characteristic of Ets (Yamada et al. 2017). Indeed, pentagalloyl glucose with two HHDP groups is also called casuarictin. The high correlation between Sanguiin H6 and Lambertianin C (R = 0.86) may reflect a precursor relationship as Sanguiin H6 can be described as a dimer of casuarictin linked through the gallic acid residue and a HHDP acid unit whereas Lambertianin C has been described as a trimer of casuarictin (Scheme 1B). The slightly less strong correlation (R = 0.76) between Sanguiin H2 and Sanguiin H6 also suggests a precursor relationship and indeed Sanguiin H6 differs from H2 by the addition of an HHDP-glucose unit (Scheme 1B). The less strong correlation (R = 0.56) between Sanguiin H6 and Sanguiin H10 may also indicate a less immediate precursor relationship as they differ by a HHDP unit.

Ellagic acid arises when HHDP groups released from Ets undergo spontaneous lactonization (Schulenburg et al., 2016). The correlation between the ET Sanguiin H2 and ellagic acid was relatively high (R = 0.72), which may indicate some metabolic connection. The correlations were less strong for the larger Ets (Sanguiin H10 (R = 0.40), Sanguiin H6 (R = 0.40) and Lambertianin C (R = 0.21) and ellagic acid, which implies a greater metabolic distance between the larger Ets and ellagic acid.

The correlations between ellagic acid and some of the ellagic acid derivatives was also interesting. There was a strong correlation between EllagicPent1 and MeEll pent (R = 0.82), which suggests a precursor relationship between these compounds. However, the correlation between EllagicPent2 and MeEll pent was negative (R = -0.29). The consensus is that the first eluting ellagic pentose is likely to be a xyloside and the latter eluting one an arabinoside (Gasperotti et al., 2010; Mullen et al., 2003). Therefore, this correlation may reflect that the pentose attached to EllagicPent1 is most probably xylose and arabinose in EllagicPent2. Indeed, there was a negative correlation between Ellagic pent 1 and 2 (R = − 0.6) with a bimodal pattern (Fig. S2C).

#### LG6

**Fig. 5:**
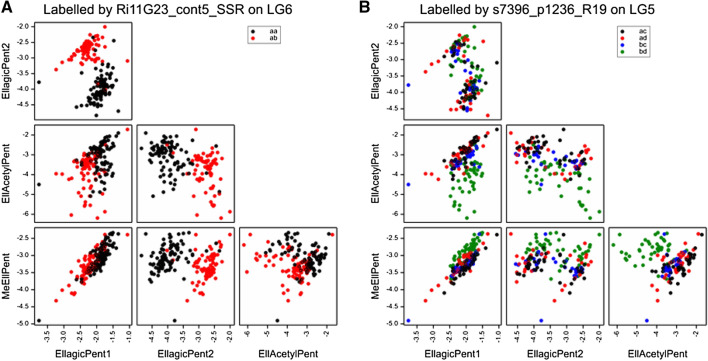
Scatterplots of the ellagic acid derivatives, labelled by markers close to the major QTLs on **A** LG6 and **B** LG5. The colour coding reflects the alleles inherited from the parents, ac, ad, bc & bd

LG6 has few markers from Glen Moy and so most QTLs are due to differences between the alleles inherited from Latham. LG6 shows a very significant area for ellagic acid derivatives close to 80 cM in each year. There was a major QTL for EllagicPent2 (arabinose) for each year (giving it a bimodal distribution), with LOD 55 for the over-years average and R^2^ of 74%. This is in the same region as a QTL for total phenol content identified in previous work (Dobson et al., 2012). EllagicPent1 (xylose) also had a major QTL here, with LOD 13.4, and R^2^ of 27%, but with the opposite effect to EllagicPent2. MeEllPent and EllAcetylPent also have strong QTLs with the same direction as EllagicPent1 (Xyl) in this region, and the flavonol Kglu has a major QTL (LOD 11.9, R^2^ 24%). Figure 5A shows a scatter plot of the over-year genotype means for the ellagic acid derivatives, labelled by marker Ri11G23_cont5_SSR at 80 cM, displaying the separation of the distribution of EllagicPent2 (Ara) between the two genotypes. The bimodal distribution for EllagicPent2 (Ara) complicates correlations; for example, the overall correlation between EllagicPent1 (Xyl) and EllagicPent2 (Ara) in 2010 was -0.60, but within each of the clusters corresponding to the genotypes of Ri11G23_cont5_SSR the correlation is positive. There was also a strong QTL for the ellagitannin Sanguiin H10 in 2010 only, with the peak LOD of 5.9 at 71 cM.

There are some genes underlying the QTL region (75–80 cM) on LG6 that could be implicated in the control of transfer of sugars to flavonoids. Once again there are homologues (> 80% homology) with *Arabidopsis* genes that encode uridine diphosphate glycosyltransferases (UDPGTs), one of which has been described as specific for the transfer of glucose to quercetin to form quercetin 7-*O*-glucoside (AT1G05675.1–rabidopsis.org) but another is described as specific for transfer to benzoic acids, including salicylates (AT2G43820.1). However, homologues in other species have not been described with these specificities so it is possible that the raspberry gene encodes a transferase that could be involved in the transfer of pentoses (arabinose or xylose) to ellagic acid.

#### LG5

LG5 also has major QTLs in the over-years analysis for ellagic acid derivatives EllAcetylPent (LOD 27.2, R^2^ 47%) and MeEllPent (LOD 10.2, R^2^ 21%) at 8–16 cM. For EllAcetylPent, offspring with genotype ac had the highest level, those with genotype ad and bc had intermediate levels and bd had the lowest level. The pattern was reversed for MeEllPent. Figure 5B shows a scatter plot of the ellagic acid derivatives, labelled by marker s7396_p1236_R19 at 17 cM on LG5. Neither EllAcetylPent nor MeEllPent have bimodal distributions, but their scatterplot shows a separation of the bd genotype (shown in green) from the other three genotypes. Once again, these metabolites have an overall negative correlation (Fig. S2C), but a positive correlation within the QTL genotype clusters. KGlu has a strong QTL nearby, at 19 cM, with a similar pattern to EllAcetylPent.

There were also strong QTLs in the over-years analysis for PelSam and PelGlu mapping to 75–76 cM on LG5, with similar effects due to the allele from Latham.

#### LG2

No major QTLs for metabolites were detected on LG2. Strong QTLs (LODs 5.7–7.3) were detected in the over-years analysis in the region 12–19 cM, for the anthocyanins CySoph, CyC_28_H_30_O_17_ and total anthocyanins. All showed a similar pattern of effects, with ac and ad having the highest levels, bc intermediate and bd lowest. A minor QTL with a similar pattern was detected nearby, at 5 cM, for QDihex2.

A second region was found at the other end of LG2 for KGlcU and QGlcU, centred at 105 cM. This matches with the position of a QTL previously identified for total phenol content (Dobson et al., 2012). However, that study was based on fewer progeny and the significance is lower. For these flavonols, genotype bc had the highest level, genotypes ac and bd were intermediate and genotype ad was lowest.

#### LG3

Unlike the other linkage groups, the QTLs on LG3 were not consistent from year to year. No QTLs for were detected here in 2015, few in 2011 but more in 2010. Therefore, results for this linkage group are given from the analysis of data from each year separately in Fig. 1 and in the discussion below.

In 2010, a strong QTL was detected for total anthocyanins at 101 cM, together with a strong QTL for CyGlu at 111 cM. This matches with a QTL for total anthocyanins noted in previous studies (Dobson et al., 2012; McCallum et al., 2010). A strong QTL for QGlu and minor QTLs for QDihex2, KGlu and Lambertianin C were also detected in the region 99–111 cM. All were predominantly Latham effects, and all had effects in the same direction, apart from Lambertianin C, which showed the opposite effect. There was also a minor QTL detected for PelGlu at 76 cM, where the significant effect was from Glen Moy. Finally, there was a minor QTL for KGlcU at 8 cM (a significant effect of Latham). In 2011, a strong QTL was detected for Sanguiin H10 at 53 cM, and minor QTLs were detected for MeEllagicPent and EllagicPent1 (presumed xyloside) at 61 cM, all with effects from both parents. Interestingly, the QTL for Sanguiin H10 overlaps a QTL for total phenol content noted previously (Dobson et al., 2012), albeit from a smaller progeny set. There was also a minor QTL at KGlu at 98 cM, an effect of Latham.

## Conclusions

Major and consistent QTLs were established for many of the polyphenol components of red raspberry, which have been previously implicated in health beneficial effects. This indicates that there is considerable genetic control of the levels of these components and provides confidence that markers could be deployed in breeding programmes to accelerate breeding of cultivars with defined polyphenol profiles. Many of these QTLs have underlying candidate genes that could influence accumulation of specific polyphenols. For example, the QTL region at 25–33 cM on LG4 had major QTLs for flavonoid components (QHexPent, QPent and CySam) which contained pentose sugars and was underlaid by a homologue of a UDP-glycosyltransferase implicated in the transfer of xylosyl groups to produce CySam from CyGlu in Arabidopsis [AT5G54060(UF3GT) (arabidopsis.org)]. This region could be examined for markers which could be used in accelerated breeding of new raspberry varieties with elevated levels of these specific anthocyanins and flavonols. Future work could examine the expression of some of the candidate genes indicated across the ripening stages of the parents (e.g., Scolari et al., 2021) to confirm if their expression matched the known accumulation of the specific polyphenols.

There was good correlation across years between various ellagic acid derivatives which suggested metabolic inter-connectivity. Similar inter-connectivity was noted for flavonol components, some following expected biosynthetic pathways but others less easily explainable. The correlations in levels of ellagitannins could reflect metabolic patterns and inter-relationships in the synthesis of these large polyphenol components. This is interesting because the biosynthetic pathways that lead to the formation of large ellagitannin structures are still not well defined despite pioneering work which suggested controlled oxidation of pentagalloyl glucose groups by specific laccases (see Niemetz & Gross, 2005). Closer examination of the genes that underly the strong QTLs for the ellagitannins, Sanguiin H6 and Lambertianin C, on LG3 at 124 cM may reveal candidate genes involved in the formation of these abundant and biologically active components. Once again, backing up with candidate gene expression studies could be interesting as ellagitannins are known to be accumulated early in the fruit formation (Beekwilder et al., 2005).

## Supplementary Information

Below is the link to the electronic supplementary material.

Supplementary file1 (DOCX 3169 KB)

## Data Availability

The metabolomic data may be made available on request to the corresponding author.
