# Supplementary material for: Quantitative trait loci mapping of polyphenol metabolites from a ‘Latham’ x ‘Glen Moy’ red raspberry (Rubus idaeus L) cross
Source: Metabolomics. 2023 Aug 8;19(8):71. doi: 10.1007/s11306-023-02033-7 (PMC10409862; doi:10.1007/s11306-023-02033-7)

**Fig. S1A PDA profiles (280 nm) of the parents**

RT:

0.00 - 12.00

0

1

2

3

4

5

6

7

8

9

10

11

12

**Time (min)**

0

500000

1000000

1500000

**μAU**

Morin IS

QGlcU

Anthocyanin peaks

Sanguiin H10

Sanguiin H6

FSD = 1.55e6

**UV 280**

**Latham**

**Moy**

Lamb C

**Fig. S1B PDA profiles at 520 nm of the parents showing differences in anthocyanin peaks A1 – A12**

RT:

0.00 - 8.00

0

1

2

3

4

5

6

7

8

**Time (min)**

0

10

20

30

40

50

60

70

80

90

100

Relative Absorbance

FSD = 1.05e5

**A520**

Latham

Moy

A1 - A4

A5 – A6

A7 - A12

**Fig S2A: Correlation Matrix of Anthocyanins**
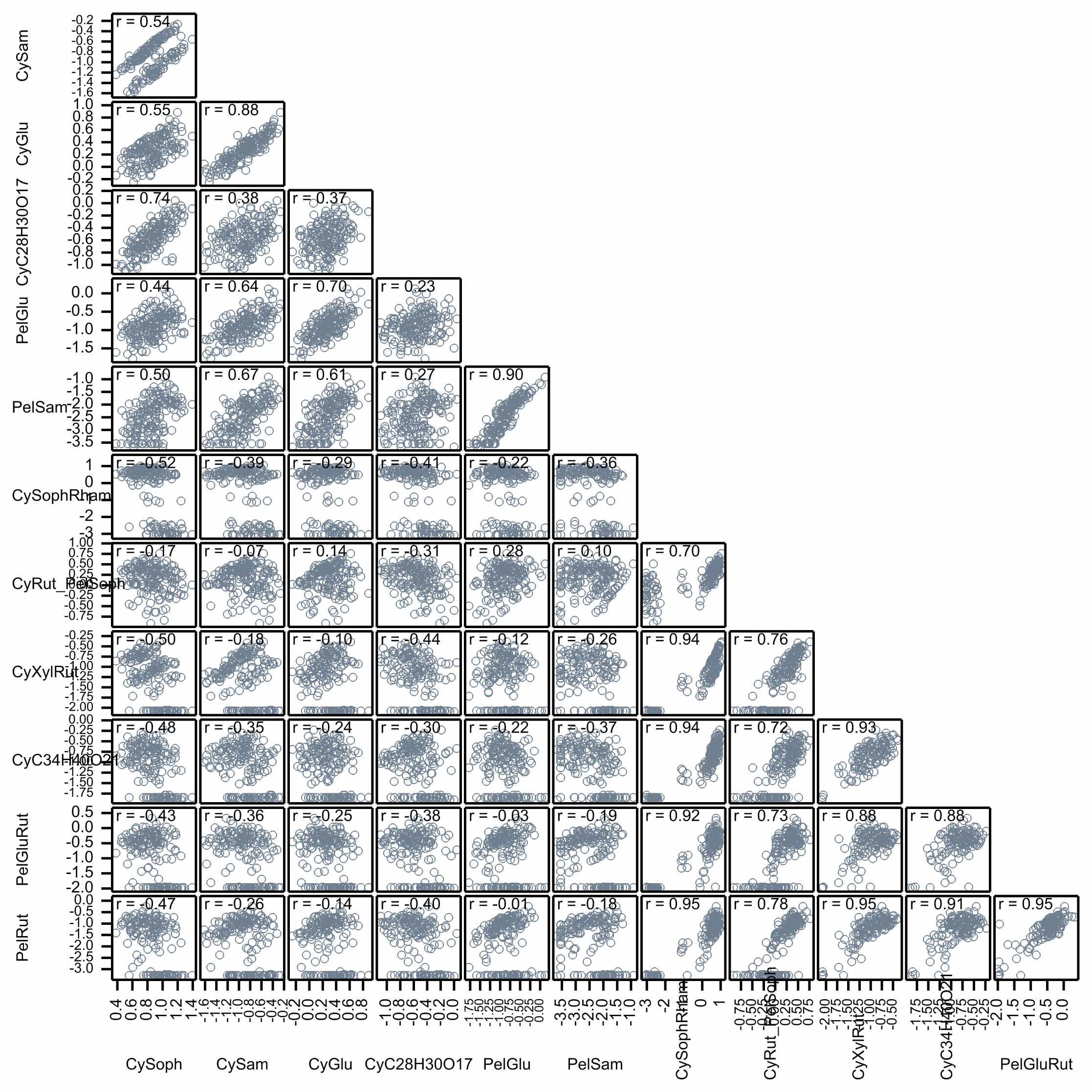


Components where the ID is not confirmed only the first possible ID is given, e.g., CyXylRut is used for CyXylRut/CySamRham. Red arrow is mentioned in the text.

**Fig S2B: Correlation Matrix of Flavonols**


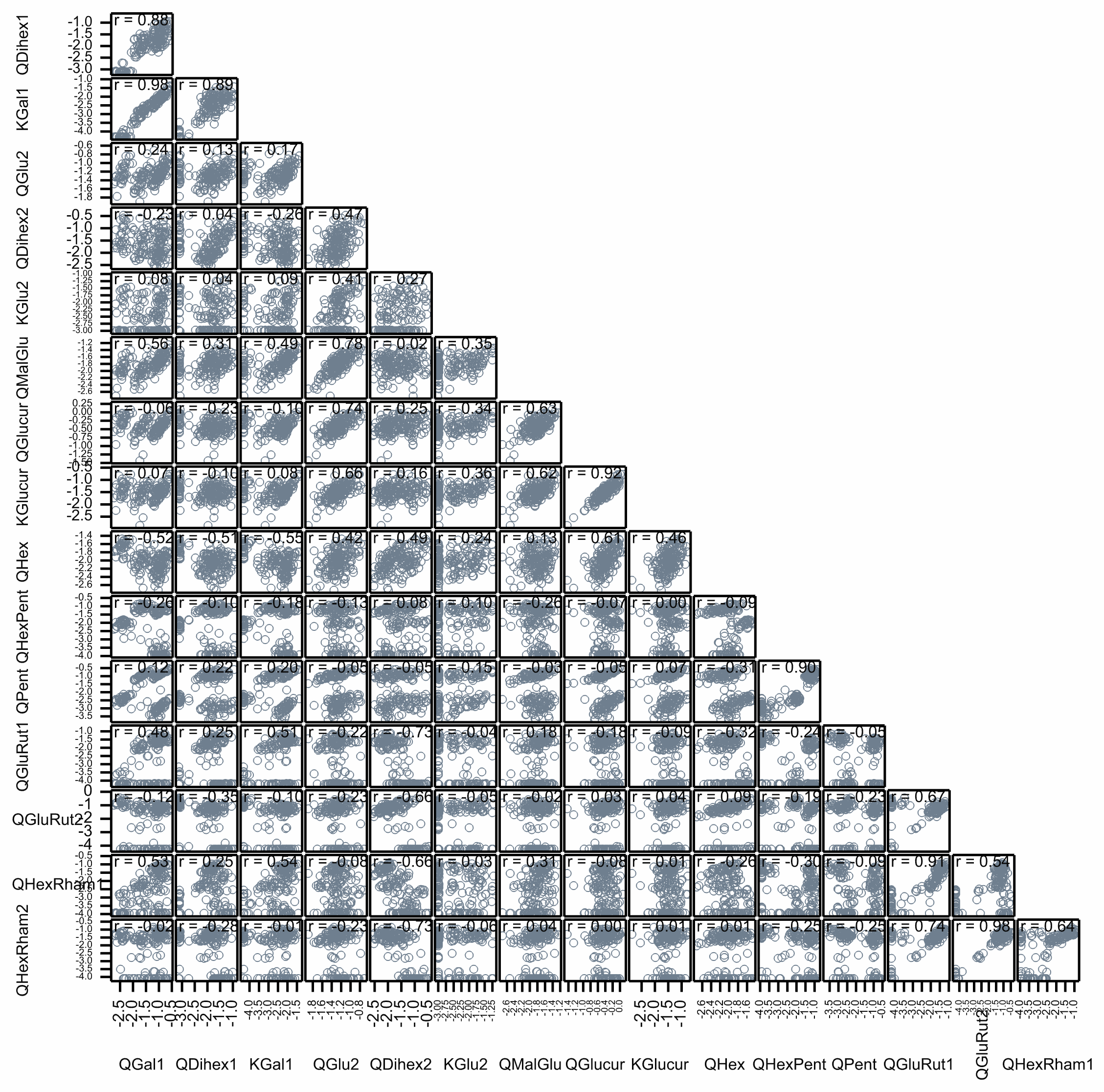


**Fig S2C: Correlation Matrix of Ellagitannins and ellagic acid derivatives**


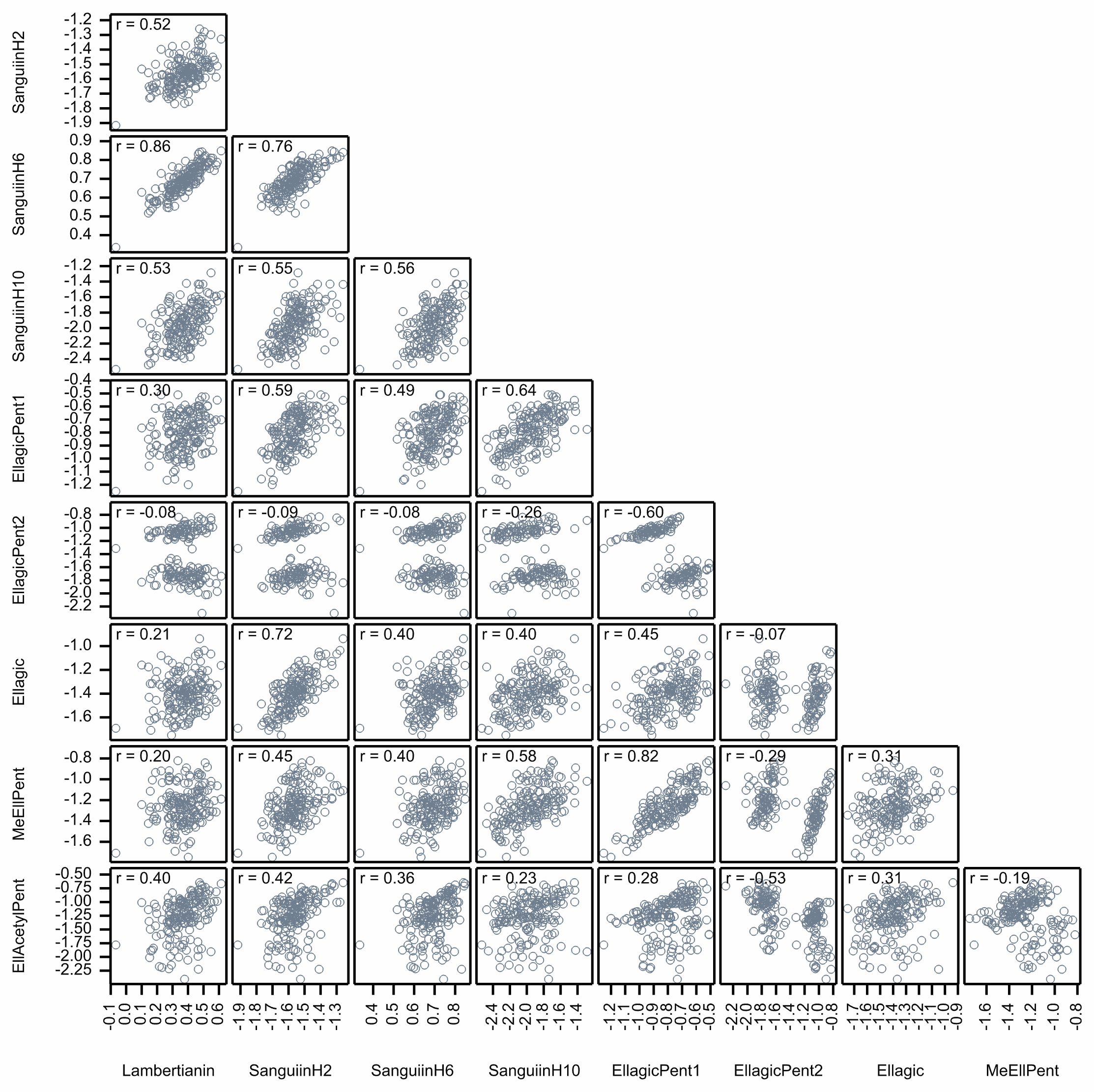


**Fig S2C: Correlation Matrix of Flavonols and Anthocyanins**


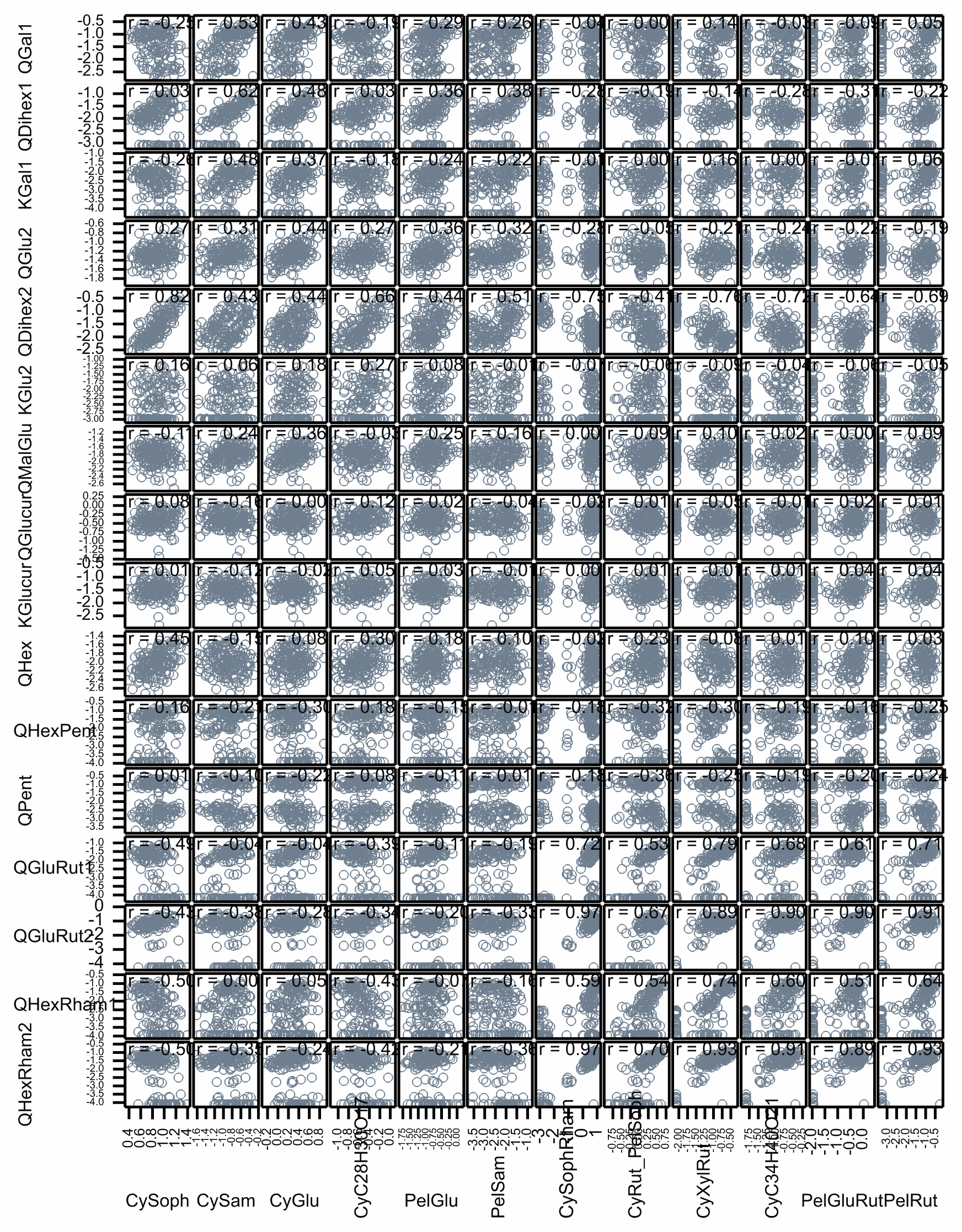

Supplement: Supplementary file 1 — Supplementary file1 (DOCX 3169 KB) [file 11306_2023_2033_MOESM1_ESM.docx]
